# Transient rapamycin treatment during developmental stage extends lifespan in *Mus musculus* and *Drosophila melanogaster*

**DOI:** 10.15252/embr.202255299

**Published:** 2022-07-07

**Authors:** Giuseppe Aiello, Cosimo Sabino, Davide Pernici, Matteo Audano, Francesco Antonica, Matteo Gianesello, Claudio Ballabio, Alessandro Quattrone, Nico Mitro, Alessandro Romanel, Alessia Soldano, Luca Tiberi

**Affiliations:** ^1^ Armenise‐Harvard Laboratory of Brain Disorders and Cancer, Department CIBIO University of Trento Trento Italy; ^2^ DiSFeB, Dipartimento di Scienze Farmacologiche e Biomolecolari Università degli Studi di Milano Milan Italy; ^3^ Laboratory of Translational Genomics, Department CIBIO University of Trento Trento Italy; ^4^ Laboratory of Bioinformatics and Computational Genomics, Department CIBIO University of Trento Trento Italy

**Keywords:** aging, early‐life treatment, mTOR, sulfotransferases, Metabolism

## Abstract

Lifespan is determined by complex and tangled mechanisms that are largely unknown. The early postnatal stage has been proposed to play a role in lifespan, but its contribution is still controversial. Here, we show that a short rapamycin treatment during early life can prolong lifespan in *Mus musculus* and *Drosophila melanogaster*. Notably, the same treatment at later time points has no effect on lifespan, suggesting that a specific time window is involved in lifespan regulation. We also find that sulfotransferases are upregulated during early rapamycin treatment both in newborn mice and in *Drosophila* larvae, and transient dST1 overexpression in *Drosophila* larvae extends lifespan. Our findings unveil a novel link between early‐life treatments and long‐term effects on lifespan.

## Graphical abstract

This study reveals a link between early‐life treatments and long‐term effects on lifespan in *Drosophila* and mice.

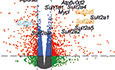

## Introduction

Genetic and environmental conditions in early organism development could influence traits later in life, including diseases, aging, and lifespan (Barker & Osmond, 1986; Ozanne & Hales, 2004; Gluckman *et al*, 2008). Indeed, few studies have reported that different diets for pregnant mothers or for young mice affect offspring survival (Ozanne & Hales, 2004; Sun *et al*, 2009). Notably, there is also controversial evidence about the correlation between early‐life treatments and lifespan extension in mammals (Ozanne & Hales, 2004; English & Uller, 2016). Similar experiments conducted in *Drosophila melanogaster and Caenorhabditis elegans* showed that dietary or changes in cellular ROS levels during development can induce lifespan extension (Stefana *et al*, 2017; Obata *et al*, 2018; Bazopoulou *et al*, 2019). Interestingly, lifespan can be also modulated through the regulation of the mTOR pathway. This signaling is evolutionarily conserved from yeast to mammals and regulates growth and metabolism in response to growth factors, amino acids, stresses, and changes in cellular energy status (Liu & Sabatini, 2020). Inhibition of the mTOR signaling pathway by genetic or pharmacological intervention extends lifespan in vertebrates, yeast, nematodes, and fruit flies (Kapahi *et al*, 2004; Kaeberlein *et al*, 2005; Fontana *et al*, 2010). Treating mice with rapamycin, an inhibitor of the mTOR pathway, from 20 months of age extends the median and maximal lifespan of both male and female mice (Harrison *et al*, 2009) and the same effect has been observed in *Drosophila melanogaster* (Bjedov *et al*, 2010). Nevertheless, most of the published data on dietary interventions and drug treatments have been performed during the adult life of several organisms (Fontana *et al*, 2010), while early‐life rapamycin administration has never been tested in wild‐type mice (Way *et al*, 2012). Indeed, transient rapamycin administration was only used in elder mice (Bitto *et al*, 2016; Strong *et al*, 2020) and the mechanisms behind this lifespan increase are elusive. Here, we investigated whether an early‐life and transient rapamycin administration can prolong lifespan in two animal models, *Mus musculus* and *Drosophila melanogaster*.

## Results

**Figure 1:**
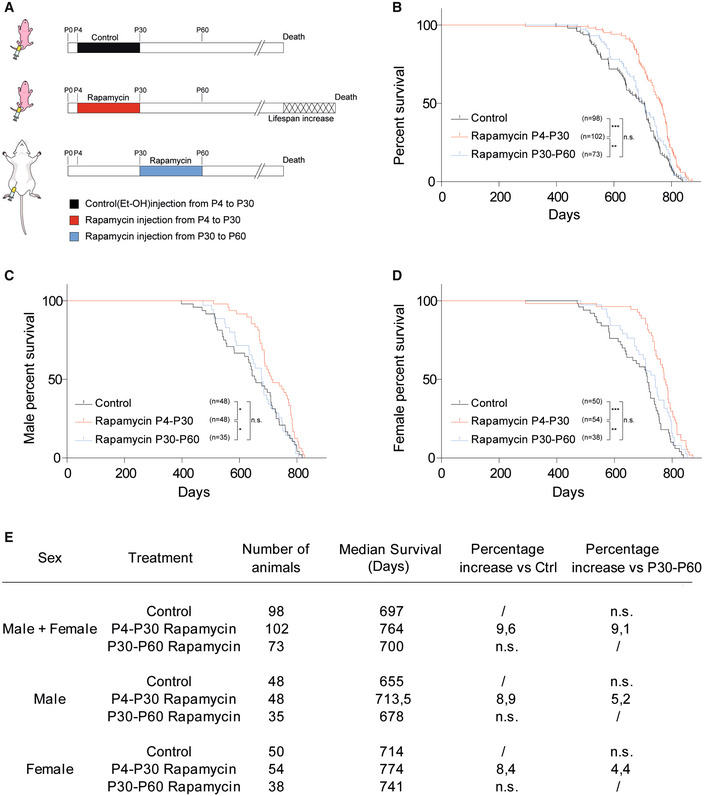
P4‐P30 rapamycin treatment increases mouse lifespan ASchematic illustration of the experimental procedure and results. Control mice were intraperitoneally injected daily with EtOH from postnatal day 4 to postnatal day 30 (P4‐P30). Treated mice were intraperitoneally injected daily with rapamycin during two distinct temporal windows, P4‐P30 or P30‐P60. P4‐P30 rapamycin‐treated mice show a lifespan increment compared with control and P30‐P60 rapamycin‐treated mice.BSurvival curves of control mice, P4‐P30 rapamycin‐treated mice, and P30‐P60 rapamycin‐treated mice including data from both sexes (males + females). Log‐rank (Mantel–Cox) test.C, DSurvival curves of male (C) and female (D) control mice, P4‐P30 rapamycin‐treated mice, and P30‐P60 rapamycin‐treated mice. Log‐rank (Mantel–Cox) test.ELog‐rank test on survival analysis (summary table).

**Figure 2:**
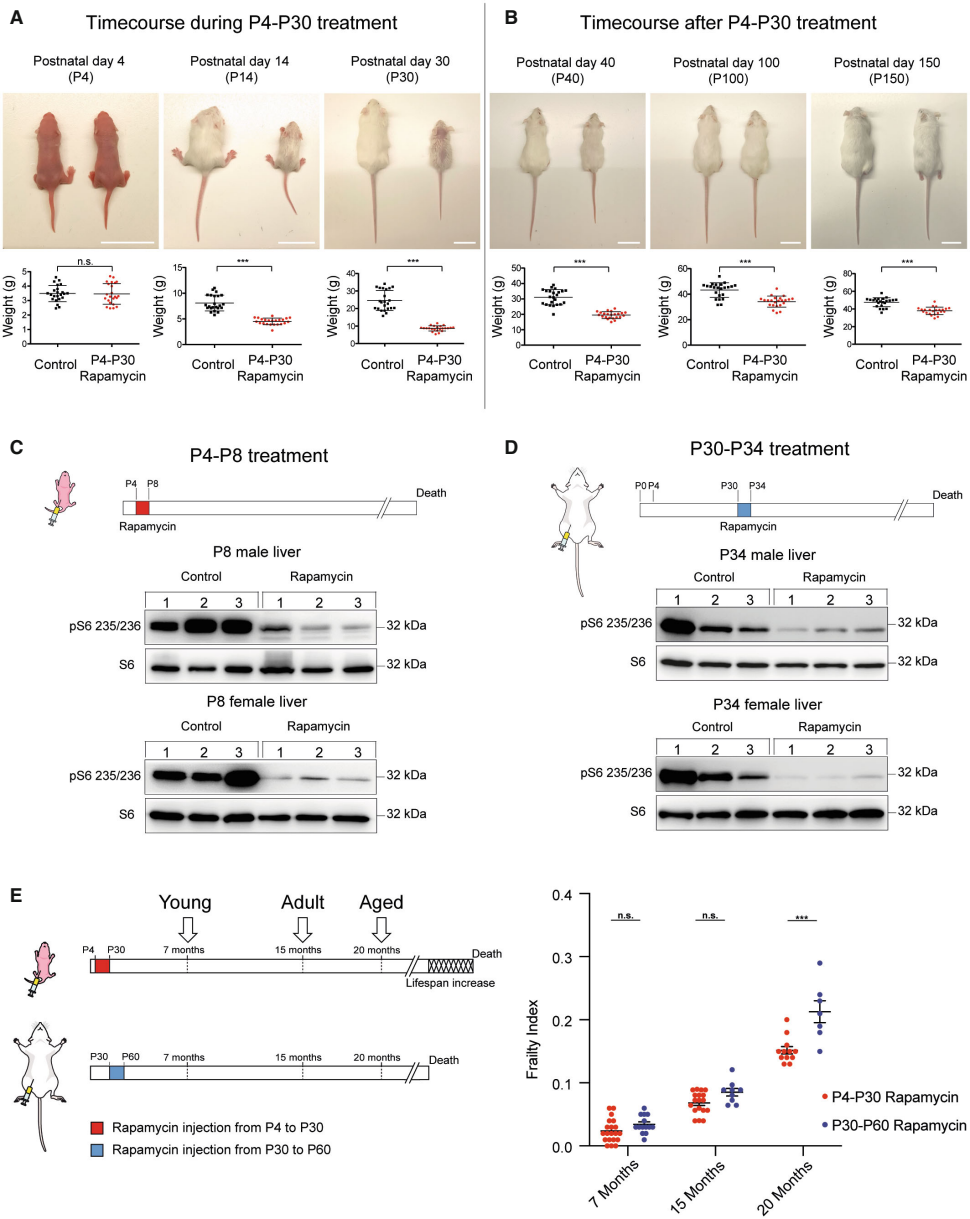
Physical and physiological status of P4‐P30 and P30‐P60 rapamycin‐treated mice AUpper panels: Representative pictures showing rapamycin effects on mouse body size during P4‐P30 treatment. Images of *n* = 23 biological replicates (both for control and for rapamycin‐treated mice) have been taken at P4, P14, and P30 (end of the treatment). Scale bar: 3 cm. Lower panels: Scatter dot plot indicating body weight of control and rapamycin‐treated mice during P4‐P30 treatment. Data are indicated as mean + SD. Two‐tailed Student's *t*‐test; ***P < 0.0005, n.s., not significant.BUpper panels: Representative pictures showing rapamycin effects on mouse body size after P4‐P30 treatment. Images of *n* = 23 biological replicates (both for control and for rapamycin‐treated mice) have been taken at P40, P100, and P150. Scale bar: 3 cm. Lower panels: Scatter dot plot indicating body weight of control and P4‐P30 rapamycin‐treated mice after the treatment. Data are indicated as mean + SD. Two‐tailed Student's *t*‐test; ***P < 0.0005, n.s., not significant.C, DUpper part, schematic illustration of the experimental procedure. Western blot analysis of S6 ribosomal protein and phospho‐S6 ribosomal protein (Ser235/236) from whole‐liver protein extracts of female and male at P8 (C) and P34 (D) rapamycin‐treated mice. Mice were sampled after 4 days of EtOH or rapamycin treatment.ESchematic illustration of the different time points analyzed (left side) and Frailty Index box (right side) of P4‐P30 and P30‐P60 rapamycin‐treated mice. Red scatter dots indicate the Frailty Index of P4‐P30 rapamycin‐treated mice at 7 months (*n* = 20; biological replicates), 15 months (*n* = 18; biological replicates), and 20 months (*n* = 12; biological replicates). Blue scatter dots indicate the Frailty Index of P30‐P60 rapamycin‐treated mice at 7 months (*n* = 14; biological replicates), 15 months (*n* = 9; biological replicates), and 20 months (*n* = 7; biological replicates). Data are indicated as mean + SEM. Two‐way ANOVA; ***P < 0.0005, n.s., not significant.

**Figure EV1:**
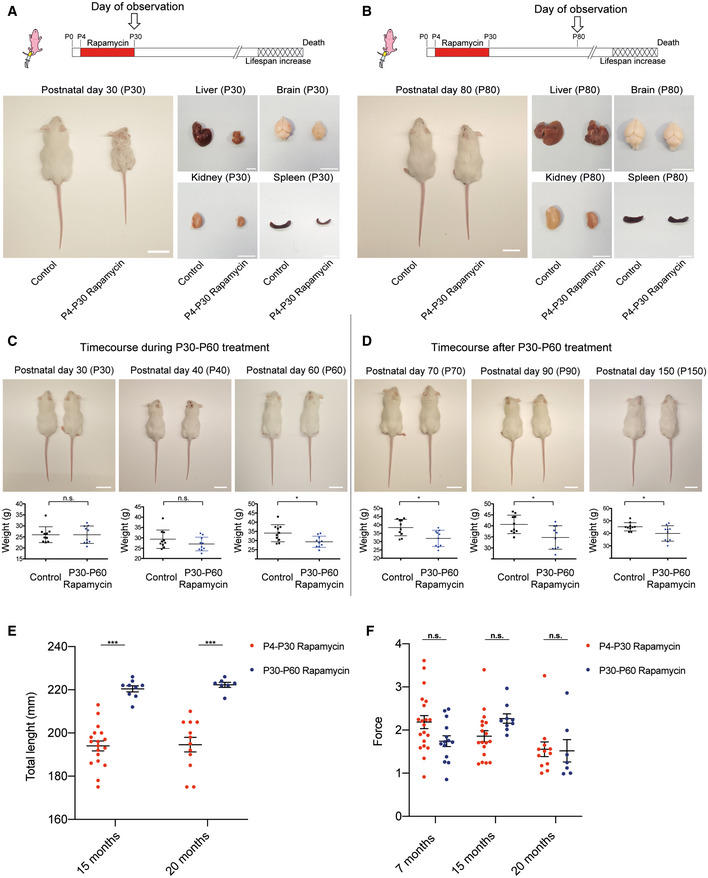
Analysis of the physical status of P4‐P30 and P30‐P60 rapamycin‐treated mice at different time points A, BUpper panels: Schematic illustration of the experimental procedures. Rapamycin effects on body and organ size in P4‐P30 rapamycin‐treated mice. Representative images of liver, brain, kidney, and spleen have been taken at P30 (A) and P80 (B). Scale bar: 3 cm.CUpper panels: Representative images showing rapamycin effects on mice body size during P30‐P60 treatment. Images of *n* = 10 biological replicates (both for control and for rapamycin‐treated mice) have been taken at P30, P40, and P60 (end of treatment). Scale bar: 3 cm. Lower panels: Scatter dot plot indicating body weight of control and P30‐P60 rapamycin‐treated mice during treatment. Data are indicated as mean + SD. Two‐tailed Student's *t*‐test; *P < 0.05, n.s., not significant.DUpper panels: Representative images showing rapamycin effects on mice body size after P30‐P60 treatment. Images of *n* = 10 biological replicates (both for control and for rapamycin‐treated mice) have been taken at P70, P90, and P150. Scale bar: 3 cm. Lower panels: Scatter dot plot indicating body weight of control and P30‐P60 rapamycin‐treated mice after treatment. Data are indicated as mean + SD. Two‐tailed Student's *t*‐test; *P < 0.05.EDot plots indicating the total length (mm) of P4‐P30 and P30‐P60 rapamycin‐treated mice. Red scatter dots indicate the measurements of P4‐P30 rapamycin‐treated mice at 15 months (*n* = 18) and 20 months (*n* = 12). Blue scatter dots indicate the measurements of P30‐P60 rapamycin‐treated mice at 15 months (*n* = 9) and 20 months (*n* = 7). Data are indicated as mean + SEM.FDot plots indicating the force (Newton/grams) resulted from grip strength analysis of P4‐P30 and P30‐P60 rapamycin‐treated mice. Red scatter dots indicate the measurements from P4‐P30 rapamycin‐treated mice at 7 months (*n* = 20), 15 months (*n* = 18), and 20 months (*n* = 12). Blue scatter dots indicate the measurements from P30‐P60 rapamycin mice at 7 months (*n* = 14), 15 months (*n* = 9), and 20 months (*n* = 7). Data are indicated as mean + SEM. Data information: Two‐way ANOVA; *P < 0.05, ***P < 0.0005, n.s., not significant.

We tested whether mouse lifespan was sensitive to early‐life modulation by performing an early‐transient rapamycin treatment on CD1 outbred mice. Rapamycin (10 mg/kg) was administered daily in two distinct temporal windows, from postnatal day 4 to postnatal day 30 (P4‐P30), or from postnatal day 30 to postnatal day 60 (P30‐P60) (Fig 1A), and the lifespan was evaluated using a Kaplan–Meier survival curve (log‐rank test). Combined data from both sexes showed a 9.6% increase in median lifespan in P4‐P30 rapamycin‐treated mice compared with control mice (treated with ethanol) and 9.1% increase compared with P30‐P60 rapamycin‐treated mice (Fig 1B and E). The analysis of each sex separately showed similar result, leading to an 8.9 and 5.2% lifespan increment in P4‐P30 rapamycin‐treated males, and 8.4 and 4.4% increment in P4‐P30 rapamycin‐treated females compared with control and P30‐P60 rapamycin‐treated mice, respectively (Fig 1C–E). Surprisingly, P30‐P60 mice did not show any significant lifespan alteration compared with control mice. This indicates that the modulation of mTOR activity could influence lifespan in a specific time window, suggesting that long‐term effects on lifespan can be determined early in life. P4‐P30 rapamycin treatment had a profound effect on mouse anatomy leading to a severe reduction in body/organ size combined with weight decrease, when compared to control mice (Figs 2A and EV1A). Nevertheless, after P30 (the end of the treatment) the treated mice underwent a rapid body weight gain, even if they did not reach the control levels (Figs 2B and EV1B). A significant but milder decrease in body weight was also detected during the P30‐P60 rapamycin treatment (Fig EV1C), and it was maintained after the treatment (Fig EV1D).

We confirmed the effectiveness of rapamycin treatment by evaluating the phosphorylation status of the ribosomal protein subunit S6 (pS6), a target substrate of S6 kinase 1 in the mTOR signaling pathway (Harrison *et al*, 2009), in P4‐P8 and P30‐P34 mouse livers (Fig 2C–D). While mTOR inhibition is of a similar extent in the two time windows, it results in different physical and physiological long‐term effects. P4‐P30 rapamycin‐treated mice remain smaller compared with their P30‐P60 counterparts throughout life, as shown by the analysis of total length at 15 and 20 months (Fig EV1E). Aging can be considered as a biological process determined by the accumulation of deficits that over time culminates in death. The analysis of different noninvasive parameters allows assessing a Frailty Index (FI) that can be used as a strong predictor of mortality and morbidity (Whitehead *et al*, 2014; Feridooni *et al*, 2015; Schultz *et al*, 2020). P4‐P30 and P30‐P60 rapamycin‐treated mice were monitored during all life, and FI was calculated at three different time points: young, 7 months old (210 days old); adults,15 months old (450 days old); and aged, 20 months old (611 days old) (Fig 2E left side). Although the two cohorts of mice showed no difference in the forelimb grip strength (Fig EV1F), we observed a significant difference in the FI at 20 months between the P4‐P30 and the P30‐P60 rapamycin‐treated mice. In fact, P30‐P60 mice showed a worst body condition score, and frequent gait disorders together with the presence of a tumor in one of seven analyzed animals that resulted in higher FI compared with the P4‐P30 rapamycin‐treated mice (Fig 2E right side and Dataset EV1). Therefore, the lifespan extension of P4‐P30 rapamycin‐treated mice is associated with amelioration in several aging traits, resulting in a better health span compared with the P30‐P60 time window. To deeper investigate the differences between the two treatments, the physical and physiological assessment has been complemented with a transcriptomic analysis of the mouse liver.

**Figure 3:**
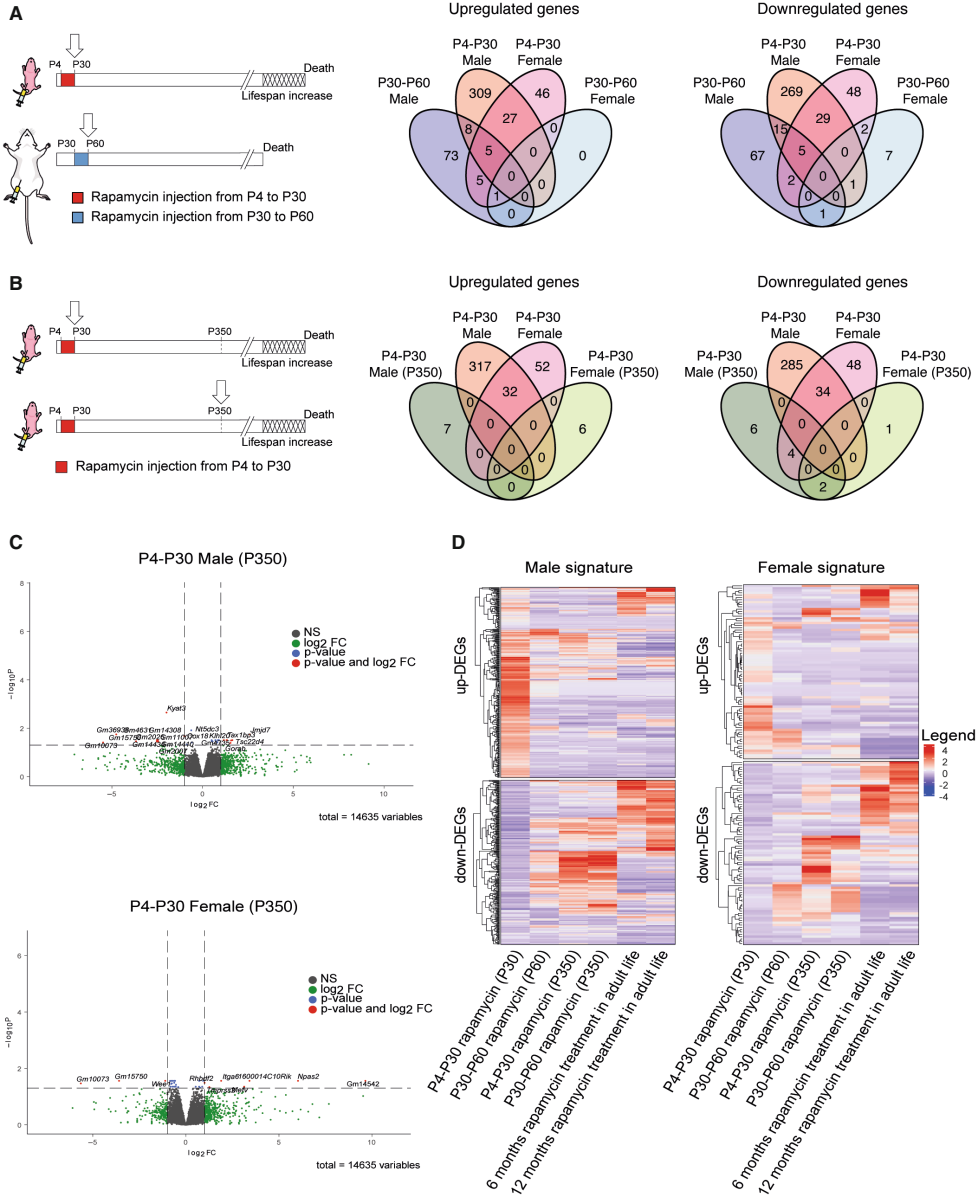
RNA‐seq analysis on P4‐P30 rapamycin‐treated mice resulted in a unique transcriptional signature ASchematic illustration of the experimental procedures (left). Landscape of up‐ and downregulated genes across P4‐P30 and P30‐P60 treatments in male and female mice. Venn diagrams are used to highlight private and shared differentially expressed genes.BSchematic illustration of the experimental procedures (left). Landscape of up‐ and downregulated genes across P4‐P30 treatment processed at the last day of treatment and at P350 in male and female mice. Venn diagrams are used to highlight private and shared differentially expressed genes.CVolcano plots showing the transcriptional changes in P4‐P30 rapamycin‐treated male (upper panel) and female (lower panel) mice processed at P350. The log2FC is represented on the *x*‐axis. The y‐axis shows the −log10 of the corrected *P*‐value. A *P*‐value of 0.05 and log2FC of 1 and −1 are indicated by gray lines. Top 10 upregulated and top 10 downregulated genes (when available) are labeled with gene symbols.DLog2FC of genes that are differentially expressed only in male (left side) and female (right side) in response to P4‐P30 treatment at the last day of treatment is compared, through heatmaps, with corresponding log2FC profiles in: P30‐P60 on the last day of treatment; P4‐P30 and P30‐P60 treatment analyzed at P350; and published data on chronic rapamycin treatment in adult life (6 and 12 months; Tyshkovskiy *et al*, 2019).

To identify genes modulated by rapamycin, several groups have analyzed the hepatic gene signature in old mice subjected to continuous rapamycin treatment (Tyshkovskiy *et al*, 2019). Indeed, the liver controls several processes (i.e., hepatic glucose, insulin signaling and lipid homeostasis) potentially implicated in lifespan regulation in mammals (Sengupta *et al*, 2010; Lamming & Sabatini, 2013). Here, to identify genes and pathways involved in the lifespan extension modulated by early‐life rapamycin treatment only, we analyzed by RNA‐seq the gene expression profiles of P4‐P30 and P30‐P60 mouse livers (sampled on the last day of treatment). Since it has been reported that rapamycin‐mediated lifespan extension is subjected to sexual dimorphism (Harrison *et al*, 2009; Bjedov *et al*, 2010), we performed differential expression analysis separately for males and females (compared against the respective control samples; see Methods). The differential analysis highlighted that females have fewer differentially expressed genes (DEGs) compared with the males (Fig 3A and Dataset EV2).

To understand the long‐term effects of early rapamycin treatments, we analyzed the transcriptome landscape of P4‐P30 and P30‐60 mice at the middle life stage (P350). Interestingly, at P350 we observed an absence of significant transcriptome differences, with only a few mildly deregulated genes (Fig 3B–C and Dataset EV3). These results confirm the concept that the inhibition of mTOR leads to different effects depending on the time of the inhibition (Baar *et al*, 2016). In addition, we investigated whether the P4‐P30 rapamycin‐associated signature correlates with the already published signature derived from chronic treatments. To do so, we compared P4‐P30 RNA‐seq data with the datasets obtained from two different chronic treatments starting at 4 months of life that differ for the administered dose of rapamycin (42 ppm and 14 ppm, respectively) and treatment duration (6 and 12 months, respectively) (Tyshkovskiy *et al*, 2019). The analysis showed that the early‐transient rapamycin treatment possesses a unique gene expression signature (Fig 3D) (compared against the respective control samples) leading us to focus on the gene expression changes on the last day of treatment.

**Figure 4:**
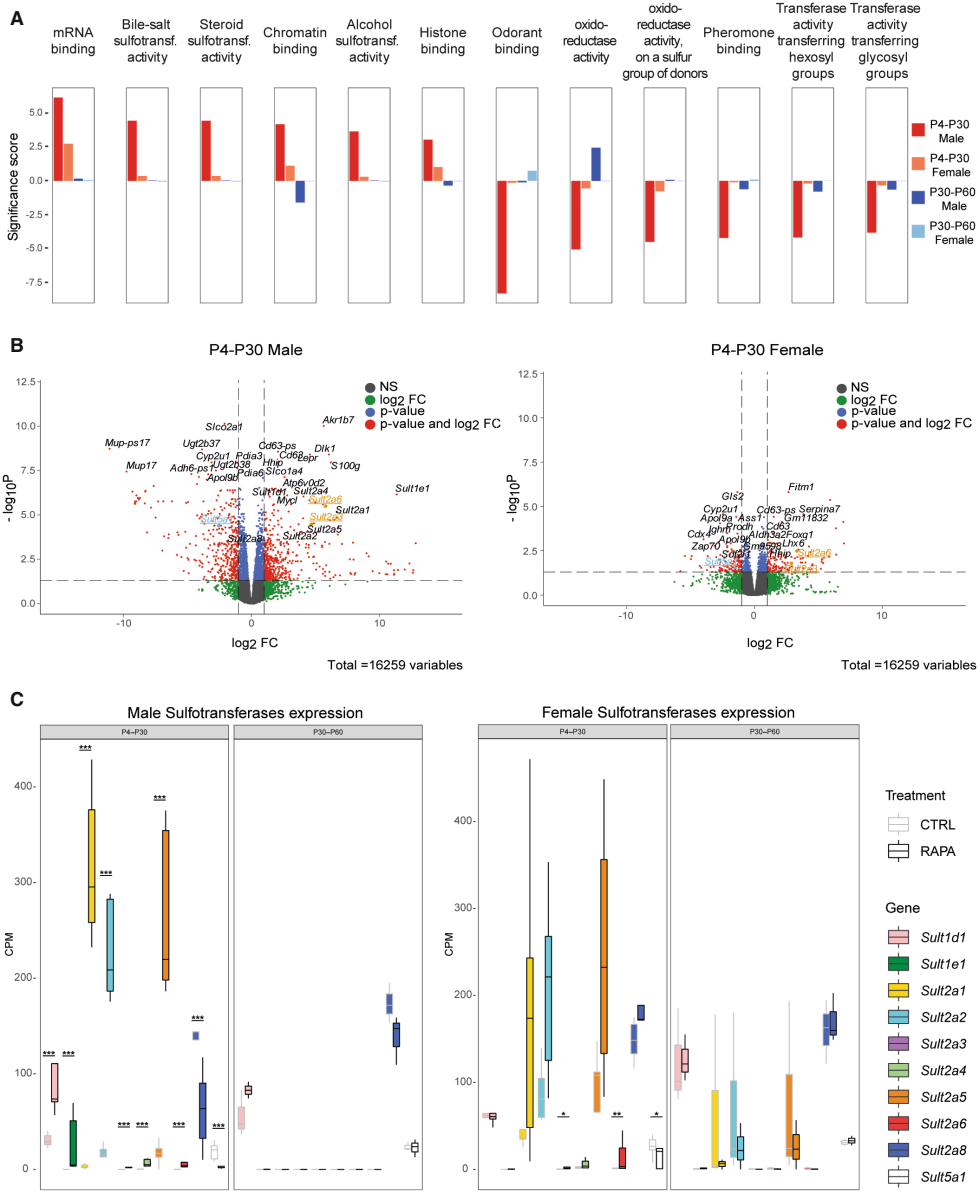
Functional enrichment analysis indicates sulfotransferase activity as a pathway involved in lifespan regulation AGene Set Enrichment Analysis (GSEA) results of P4‐P30 and P30‐P60 at the last day of treatment in male and female mice. Significance score, calculated as ‐log10(q‐value) corrected by the sign of regulation, is plotted on the y‐axis. Plots are representative of the top 12 GO Molecular Function (MF) terms with higher/lower significance scores for the male P4‐P30 rapamycin‐treated mice (top 6 with higher significance scores and top 6 with lower significance scores). The whole list of enriched GO terms is available in Dataset EV4.BVolcano plots showing the transcriptional changes in P4‐P30 rapamycin‐treated male (left side) and female (right side) mice. Each circle represents a gene. Underlined and highlighted terms are common *SULT* genes shared by males and females (orange for upregulated genes, blue for downregulated genes). The log2FC is represented on the *x*‐axis. The *y*‐axis shows the −log10 of the corrected *P*‐value. A *P*‐value of 0.05 and log2FC of 1 and −1 are indicated by gray lines. Top 10 upregulated and top 10 downregulated genes are labeled with gene symbols.CExpression profile of sulfotransferases deregulated only in the P4‐P30 time window in male (left side) and female (right side) rapamycin‐treated mice. Values for treated (dark borders) and control (light borders) samples across the different conditions are shown as median CPM with bars representing standard deviations across the five biological replicates. *P*‐values were generated by the edgeR DEG analysis, detailed in the Methods. *P < 0.05, **P < 0.005, and ***P < 0.0005.

**Figure EV2:**
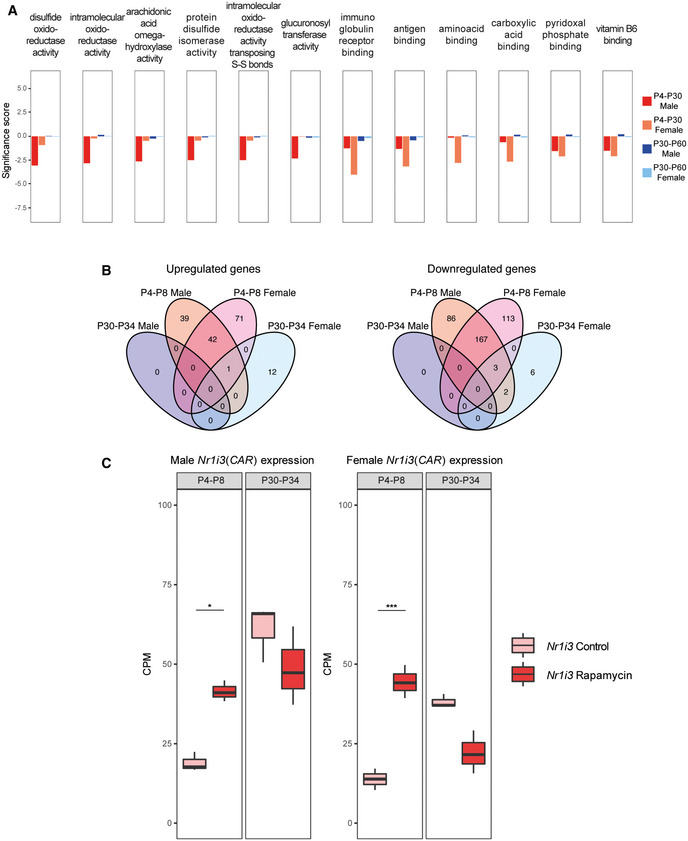
RNA‐seq analysis of acute rapamycin treatments (P4‐P8 and P30‐P34) AGene Set Enrichment Analysis (GSEA) results of P4‐P30 and P30‐P60 treatments in male and female mice. Significance score, calculated as log10 (q‐value) corrected by the sign of regulation, is plotted on the y‐axis. The whole list of enriched GO terms is available in Dataset EV4.BLandscape of up‐ and downregulated genes across the P4‐P8 and P30‐P34 treatments in male and female mice. Venn diagrams are used to highlight private and shared differentially expressed genes.CExpression profile of *Nr1i3* (*CAR*) gene across P4‐P8 and P30‐P34 male (left side) and female (right side) rapamycin‐treated mice. Values in treated and control samples across the different conditions are shown as median CPM with bars representing standard deviations across the biological replicates. Data distribution is presented through boxplots, where the central bar represents the median, while the lower and upper hinges correspond to the first and third quartiles (the 25^th^ and 75^th^ percentiles). The upper/lower whisker extends from the hinge to the largest/smallest value no further than 1.5 * IQR from the hinge (where IQR is the interquartile range, or distance between the first and third quartiles). *P*‐values were generated by the edgeR DEG analysis, detailed in the Methods. *P < 0.05, ***P < 0.0005, n.s., not significant.

To identify pathways associated with the altered gene signatures, we performed Gene Set Enrichment Analysis (GSEA) (Figs 4A and EV2A). The analysis revealed that the two time windows of treatment lead to different and at times divergent gene set enrichments (Fig 4A). For example, P4‐P30 and P30‐P60 treatments show opposite effects on chromatin binding and oxidoreductase activity (Figs 4A and EV2A and Dataset EV4). Overall, the analysis highlighted the broad upregulation of the sulfotransferase gene family by rapamycin treatment. In fact, we observed an enrichment of several molecular functions related to sulfotransferase activity (steroid, bile salt, and alcohol sulfotransferase activity) in P4‐P30 rapamycin‐treated males that is not present in the P30‐P60 time window (Fig 4A). Interestingly, among the common deregulated genes in P4‐P30 males and females, the sulfotransferases *Sult2a3* and *Sult2a6* emerged as two of the upregulated genes and *Sult5a1* as downregulated gene (Fig 4B and Dataset EV2). Moreover, several sulfotransferases (SULTs), such as *Sult1d1*, *Sult1e1*, *Sult2a1*, *Sult2a2*, *Sult2a3*, *Sult2a4*, *Sult2a5*, *Sult2a6*, *Sult2a8*, and *Sult5a1*, are upregulated only in the P4‐P30 time window (Fig 4C). Sulfotransferases are a supergene family of enzymes involved in sulfonate conjugation processes, catalyzing the transfer of sulfonate (SO_3_^−^) to a hydroxyl or amino group. Hepatic regulation and activity of SULTs could vary based on age and sex (Kocarek *et al*, 2008), and their expression is controlled by numerous members of the nuclear receptor (NR) superfamily, that act as sensors of xenobiotics and endogenous molecules, such as fatty acids, bile acids, and oxysterols (Runge‐Morris *et al*, 2013).

**Figure 5:**
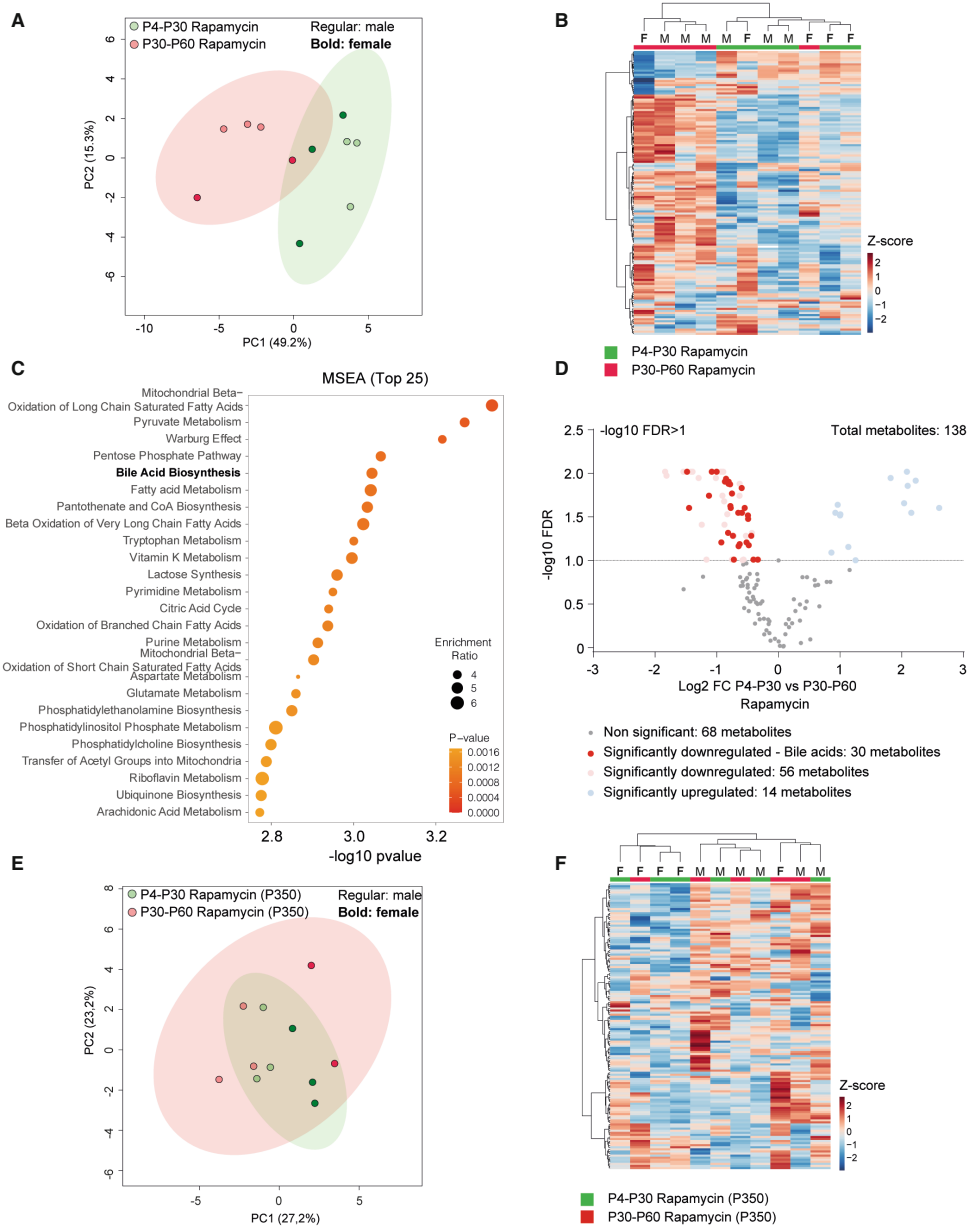
Early‐transient treatment with rapamycin increases bile acid liver detoxification in mice A, BPrincipal component analysis (PCA) (A) and heatmap (B) of liver metabolomic profile from P4‐P30 (green samples) and P30‐P60 (red samples) mice treated with rapamycin.CTop 25 metabolic pathways enriched in P4‐P30 compared with P30‐P60 mice treated with rapamycin. Metabolic Set Enrichment Analysis (MSEA) was performed taking advantage of MetaboAnalyst 5.0 webtool interrogating the KEGG database. The *x*‐axis shows the −log10 of *P‐*value.DVolcano plots showing the metabolomic changes in P4‐P30 compared with P30‐P60 mice treated with rapamycin. Each circle represents one metabolite. The log2 fold change is represented on the *x*‐axis. The *y*‐axis shows the −log10 of the false discovery rate (FDR). A FDR of 0.1 is indicated by gray line. Gray, pink, and light blue dots represent unchanged, significantly downregulated, and significantly upregulated metabolites, respectively. Red dots represent significantly downregulated bile acids in P4‐P30 compared with P30‐P60 mice treated with rapamycin.E, FPrincipal component analysis (PCA) (E) and heatmap (F) of liver metabolomic profile from P4‐P30 (green samples) and P30‐P60 (red samples) mice transiently treated with rapamycin and analyzed at P350. F, female; M, male.

**Figure EV3:**
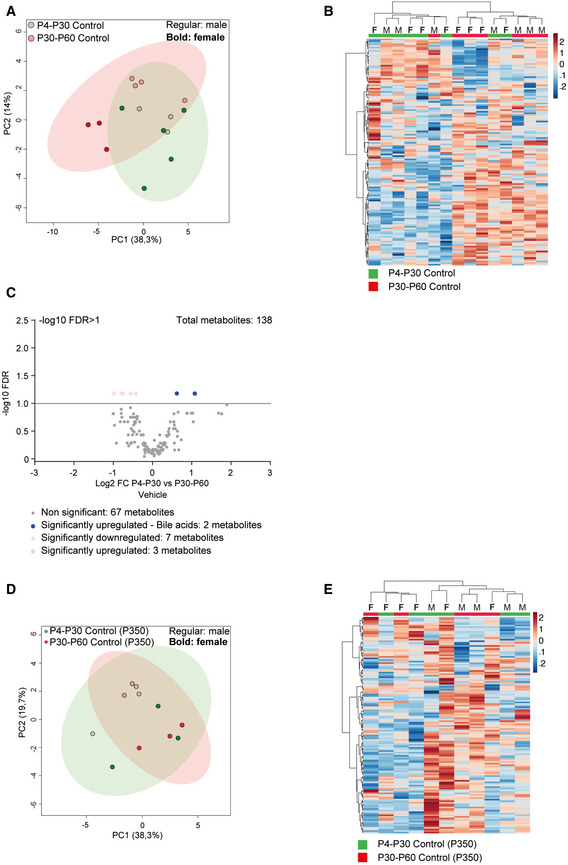
Metabolic changes in early‐transient control mice A, BPrincipal component analysis (PCA) (A) and heatmap (B) of liver metabolomic profile from P4‐P30 (green samples) and P30‐P60 (red samples) mice treated with vehicle.CVolcano plots showing the metabolomic changes in P4‐P30 compared with P30‐P60 mice treated with vehicle. Each circle represents one metabolite. The log2 fold change is represented on the x‐axis. The y‐axis shows the ‐log10 of the FDR. A FDR of 0.1 is indicated by gray line. Gray, pink, and light blue dots represent unchanged, significantly downregulated, and significantly upregulated metabolites, respectively. Blue dots represent significantly upregulated bile acids in P4‐P30 compared to P30‐P60 mice treated with vehicle.D, EPrincipal component analysis (PCA) (D) and heatmap (E) of liver metabolomic profile from P4‐P30 (green samples) and P30‐P60 (red samples) mice transiently treated with vehicle and analyzed at P350. F, female; M, male.

To determine whether the functional enrichment in sulfotransferase activity can be linked to metabolic remodeling, we performed hepatic metabolomic analysis on P4‐P30 and P30‐P60 rapamycin‐treated mice. A total of 138 metabolites were detected in the P4‐P30 and P30‐P60 time windows. We identified 70 metabolites that were significantly different across the two treatments (Dataset EV5). The P4‐P30 rapamycin treatment revealed a unique metabolic signature (Fig 5A and B) and the deregulation of several primary and conjugated bile acids (Dataset EV5). Of notice, bile acid biosynthesis was identified as one of the most enriched and significant pathways by the Metabolic Set Enrichment Analysis (MSEA) (Fig 5C). Most of the detected primary and conjugated bile acids are downregulated in P4‐P30 rapamycin‐treated mice compared with the P30‐P60 time window, suggesting the upregulation of the detoxifying pathway (Fig 5D). This observation is consistent with previous findings where higher detoxification capacity was associated with reduced intrahepatic bile acid levels (Collino *et al*, 2018). The same analysis on P4‐P30 and P30‐P60 control mice did not show the same results, indicating only a few deregulated metabolites and a stronger overlap between the two time windows (Fig EV3A–C and Dataset EV5). Interestingly, the metabolic differences revealed on the last day of treatment are not maintained later in life. Indeed, the same analysis on P4‐P30 and P30‐P60 at P350 did not reveal differences across the two treatments, showing overlap among the two time windows (Figs 5E and F, EV3D and E, and Dataset EV5). Together, these results suggest that metabolic data correlate with the transcriptome analysis, leading to focus on the changes at the last day of treatment, which reflect the direct effect of lifespan‐extending intervention, and indicating sulfotransferase activity as an important process affecting the early and transient rapamycin treatment at P4‐P30.

To clarify the role of rapamycin in the modulation of sulfotransferases, we decided to investigate the gene expression profiles of more acute treatments, such as P4‐P8 and P30‐P34. The inhibition of the mTOR pathway has a stronger transcriptome effect early in life (P4‐P8), while only a few genes are deregulated in expression when the treatment occurs later (P30‐P34) (Fig EV2B). Interestingly, the nuclear hormone receptor *Nr1i3* (*CAR*) is upregulated in both male and female P4‐P8 (Fig EV2C and Dataset EV2). CAR regulates the expression of several sulfotransferases (Runge‐Morris *et al*, 2013), and its upregulation upon rapamycin treatment might explain the enrichment of this category of genes in the P4‐P30 rapamycin‐treated mice. These data strongly support the idea that rapamycin has different effects based on the age of administration and suggest that the sulfotransferase pathway may be involved in lifespan regulation.

**Figure 6:**
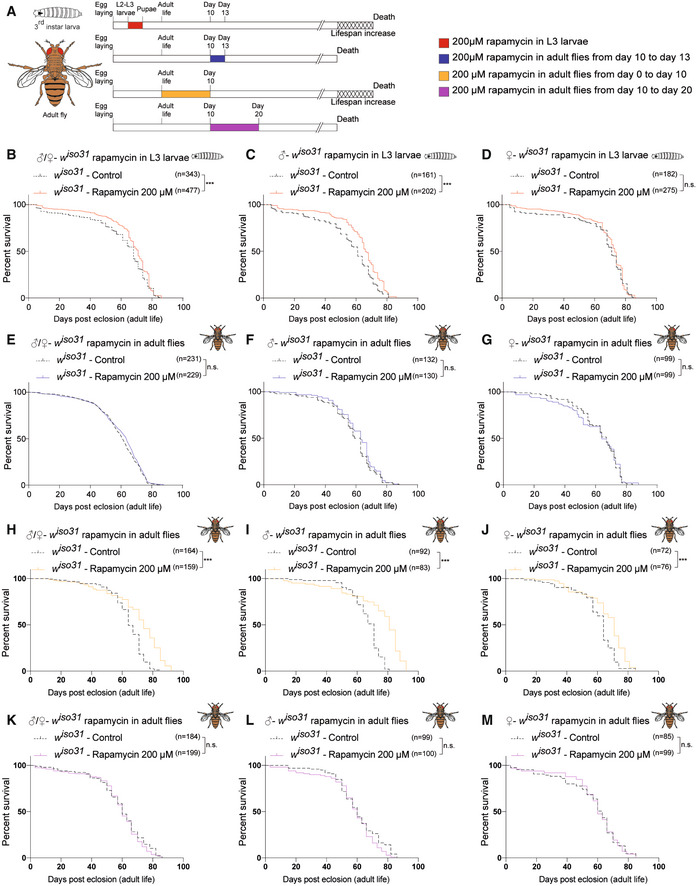
Early‐transient rapamycin treatment on *w* ^*iso31*^ *Drosophila melanogaster* induces a time‐dependent effect on lifespan ASchematic illustration of the experimental procedure and results. Flies were transiently treated during larval stages with rapamycin 200 μM starting from 72 h after egg laying to puparium formation (red) or during adulthood, from day 0 to day 10 (orange), from day 10 to day 13 (blue), or from day 10 to day 20 (purple). Rapamycin administration during development and during the first 10 days of life, but not at later time points, leads to lifespan increment.BSurvival curves of *w* ^*iso31*^ flies transiently treated from 72 h after egg laying till puparium formation (males + females) with EtOH (control) or rapamycin 200 μM.C, DSurvival curves of male (C) and female (D) *w* ^*iso31*^ flies transiently treated from 72 h after egg‐laying till puparium formation with EtOH (control) or rapamycin 200 μM.ESurvival curves of *w* ^*iso31*^ flies transiently treated in adult stage, from day 10 to 13 (males + females), with EtOH (control) or rapamycin 200 μM.F, GSurvival curves of male (F) and female (G) *w* ^*iso31*^ flies transiently treated in adult stage, from day 10 to 13, with EtOH (control) or rapamycin 200 μM.HSurvival curves of *w* ^*iso31*^ flies transiently treated from day 0 to day 10 (males + females) with EtOH (control) or rapamycin 200 μM.I, JSurvival curves of male (I) and female (J) *w* ^*iso31*^ flies transiently treated from day 0 to day 10 with EtOH (control) or rapamycin 200 μM.KSurvival curves of *w* ^*iso31*^ flies transiently treated from day 10 to 20 (males + females), with EtOH (control) or rapamycin 200 μM.L, MSurvival curves of male (L) and female (M) *w* ^*iso31*^ flies transiently treated from day 10 to 20, with EtOH (control) or rapamycin 200 μM. Data information: Log‐rank (Mantel–Cox) test. ***P < 0.0005, n.s., not significant.

**Figure EV4:**
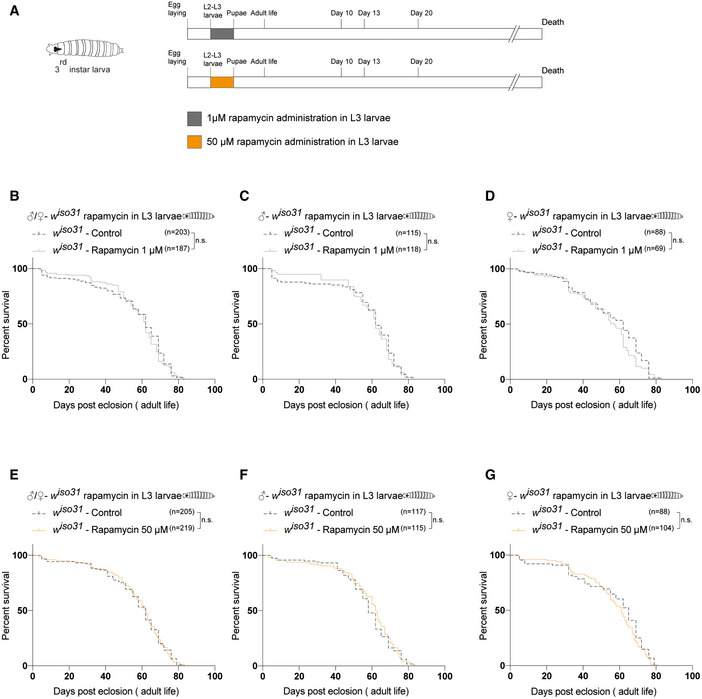
Early‐transient 1 μM and 50 μM rapamycin treatment does not induce a time‐dependent effect on lifespan ASchematic illustration of the experimental procedure and results. Flies were transiently treated during larval stages from 72 h after egg laying to puparium formation with 1 μM (gray) or 50 μM rapamycin (yellow).BSurvival curves of *w* ^*iso31*^ flies transiently treated from 72 h after egg laying until puparium formation (males + females) with EtOH (control) or rapamycin 1 μM.C, DSurvival curves of male (C) and female (D) *w* ^*iso31*^ flies transiently treated from 72 h after egg laying until puparium formation with EtOH (control) or rapamycin 1 μM.ESurvival curves of *w* ^*iso31*^ flies transiently treated from 72 h after egg laying until puparium formation (males + females) with EtOH (control) or rapamycin 50 μM.F, GSurvival curves of male (F) and female (G) *w* ^*iso31*^ flies transiently treated from 72 h after egg laying until puparium formation with EtOH (control) or rapamycin 50 μM. Data information: Log‐rank (Mantel–Cox) test; n.s., not significant.

**Figure EV5:**
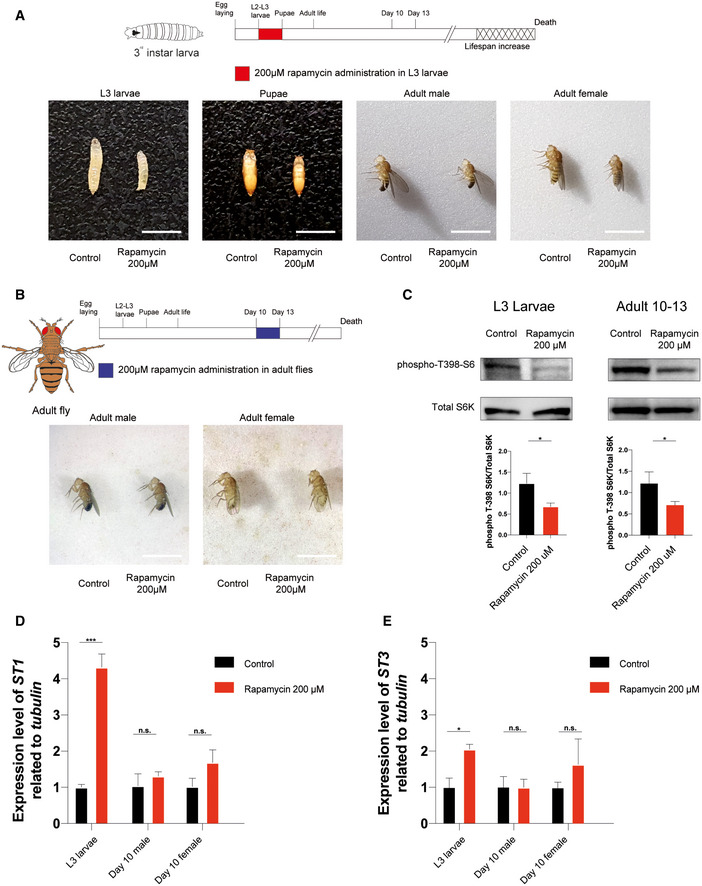
Physical status and biochemical effectiveness of rapamycin treatment in *w* ^*iso31*^ *Drosophila melanogaster* ARepresentative images showing rapamycin effects on *Drosophila* body size during and after treatment on third‐instar (L3) larvae. Images acquired during L3 larvae (120 h after egg laying), pupae, and adult (1 day post‐eclosion) stages. Scale bar: 3 mm.BRepresentative images showing rapamycin effects on *Drosophila* body size after treatment on 10‐day‐old flies. Images acquired during adult stage (3 days post‐treatment). Scale bar: 3 mm.CWestern blot analysis and quantification of S6 ribosomal protein and phospho‐T398‐S6 ribosomal protein on whole‐fly protein extracts of L3 larvae (left side) and 10‐day‐old flies treated for 3 days (right side). Two‐tailed Student's *t*‐test; *P < 0.05.D, EGene expression analysis via qRT–PCR of *dST1*(A) and *dST3*(B) in three biological replicates of *w* ^*iso31*^ L3 wandering larvae (*n* = 30 per biological replicate) and 10‐day‐old flies (*n* = 10 per biological replicate) treated with 200 μM rapamycin, 12 h after treatment. Data are indicated as mean + SEM. Two‐tailed Student's *t*‐test; *P < 0.05, ***P < 0.0005, n.s., not significant.

The discovery that lifespan can be regulated in the early stage of life in mice prompted us to investigate whether this process was evolutionarily conserved. We recreated similar experimental conditions as in mice, by administering rapamycin during larval development of *Drosophila melanogaster*. *Drosophila* life cycle can be divided into 4 developmental stages: embryo, larva (three instar stages), pupa, and adult, which correspond to four distinct periods of life: embryonic development, a juvenile growth phase, sexual maturation, and reproductive adulthood, respectively (Robertson, 1936). *Drosophila* growth occurs mainly during the juvenile larval stages, and the transition between the second (L2) and third larval instar (L3) represents an important time window during which the animal reaches the “critical weight” to continue the development (Tennessen & Thummel, 2011). For this reason, rapamycin treatment was carried out in the isogenic *white iso31* (herein *w* ^*iso31*^) *Drosophila* strain during the third‐instar larva stage. *w* ^*iso31*^ third‐instar larvae were treated with 1 μM, 50 μM, or 200 μM rapamycin, starting from 72 h after egg laying till pupal stage (Fig 6A upper timeline and Fig EV4A upper timeline). Although flies exposed to 1 μM and 50 μM did not show a significant increase in lifespan for both sexes (Fig EV4B–G and Dataset EV6), treatment with 200 μM rapamycin led to an increase in lifespan compared with the control (Fig 6B). As observed in mice, the treatment led to a reduction in body size that is maintained during all the developmental stages (Fig EV5A). Early‐transient rapamycin treatment on *Drosophila* larvae determined a significant increase in median lifespan when both sexes were analyzed together (Dataset EV6), while when the sexes were analyzed separately, only male flies showed a significant lifespan extension compared with control flies (Fig 6B–D). To verify whether the modulation of mTOR activity influences *Drosophila melanogaster* lifespan only in a specific time window as for the mammalian counterpart (Fig 1A and B), we tested the effect of rapamycin treatment at later time point. When 10‐day‐old *w* ^*iso31*^ flies were treated with 200 μM rapamycin for 3 days (Fig 6A lower timeline), no effect was observed in terms of body size and lifespan compared with control (Figs 6E–G and EV5B). The effectiveness of rapamycin treatment in the two time windows was evaluated by profiling the phosphorylation status of S6K by Western blot analysis, using a phospho‐Thr398‐dependent S6K antibody (Bjedov *et al*, 2010). Flies exposed to 200 μM rapamycin at the last day of treatment (wandering larvae and 13 days old, respectively) showed a comparable reduction in phospho‐T398‐S6K, suggesting that TOR signaling is similarly downregulated in the two time windows (Fig EV5C). These results strengthen the idea that lifespan can be determined by transient early‐life events and that similar treatments at later time points have no effect on lifespan. Although the mechanism is evolutionarily conserved, the development of *Drosophila* and mouse is significantly different and difficult to compare. To investigate whether TOR inhibition could influence *Drosophila* lifespan during other time windows, we treated *w* ^*iso31*^ flies with 200 μM rapamycin during early stages of adult life, such as the first 10 days after eclosion (adult emergence from the pupal case) (Fig 6A). Interestingly, rapamycin‐treated flies displayed an increase in lifespan compared with the control (Fig 6H), leading to an increment in the median lifespan for both sexes (Fig 6I and J and Dataset EV6). Moreover, to verify that the effect was due to the specific time window of administration and not to the duration of the treatment, we also treated 10‐day‐old *w* ^*iso31*^ flies with 200 μM rapamycin for 10 days (Fig 6A) and we observed no effect on lifespan (Fig 6K–M and Dataset EV6). In conclusion, our results indicate that inhibition of TOR in specific time windows early in life is an evolutionarily conserved mechanism that leads to lifespan extension.

**Figure 7:**
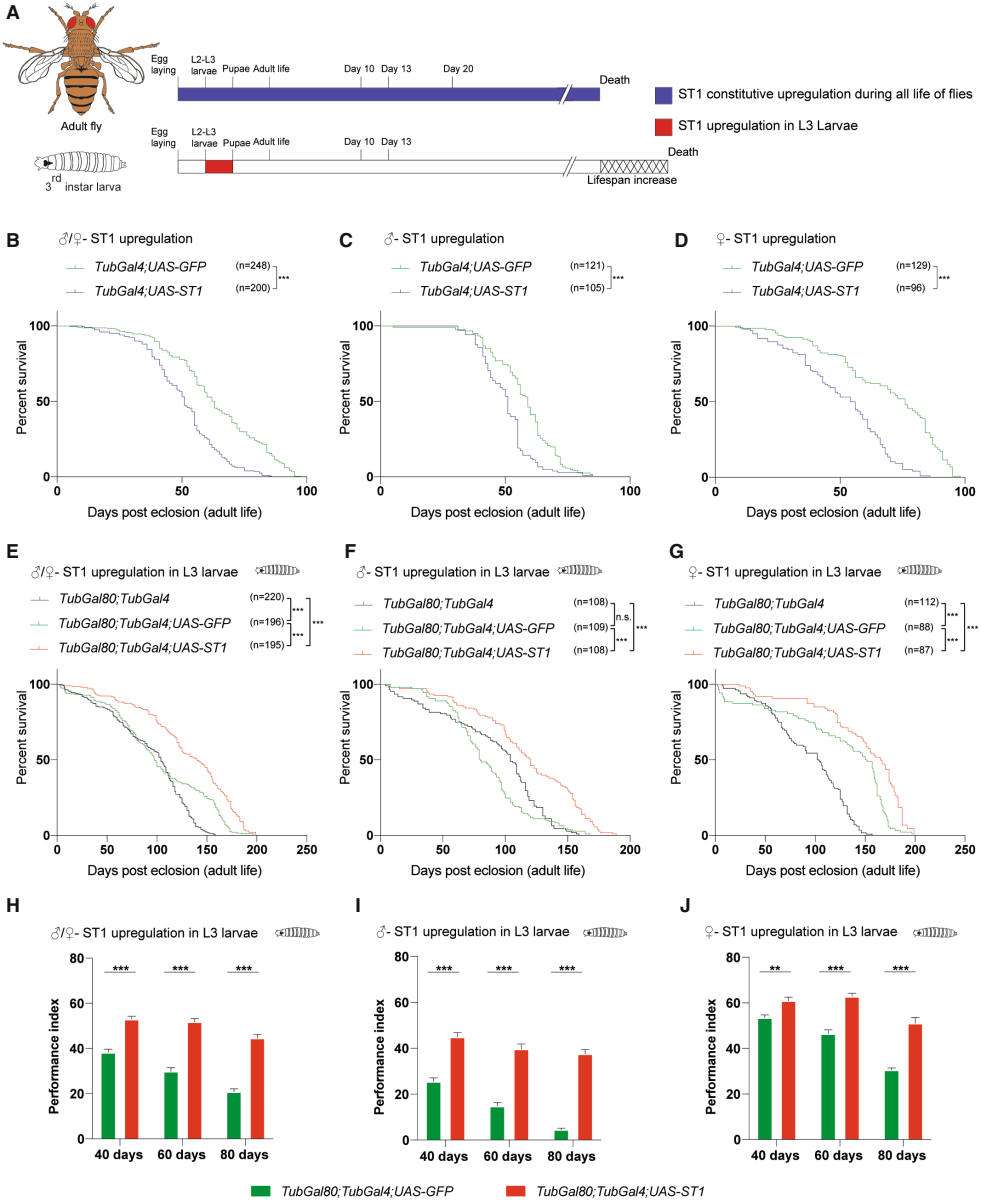
Transient dST1 overexpression during larval stage, determines an increase in *Drosophila* m*elanogaster* lifespan ASchematic illustration of the experimental procedure and results. Constitutive dST1 overexpression during the entire life (blue) of flies does not increase lifespan. Transient dST1 overexpression during larval stage (red) leads to lifespan increment.BSurvival curves of flies harboring constitutive dST1 upregulation (*TubGal4*/*UAS‐dST1)* compared with control strain (*UAS‐GFP;TubGal4*/+*)* males + females. Log‐rank (Mantel–Cox) test, ***P < 0.0005.C, DSurvival curves of flies harboring constitutive dST1 upregulation (*TubGal4*/*UAS‐dST1)* in male (C) and female (D) compared with control strain (*UAS‐GFP;TubGal4*/+*)*. Log‐rank (Mantel–Cox) test, ***P < 0.0005.ESurvival curves of flies upon upregulation of dST1 during larval stages (*tubGal80* ^*TS*^/+*;TubGal4*/*UAS‐dST1)* compared with the control strain *tubGal80* ^*TS*^/*UAS‐GFP;TubGal4*/+ (males + females) and the parental control *tubGal80* ^*TS*^ *;TubGal4* (males + females). Log‐rank (Mantel–Cox) test, ***P < 0.0005.F, GSurvival curves of male (F) and female (G) flies upon upregulation of dST1 during larval stages in (*tubGal80* ^*TS*^/+*;TubGal4*/*UAS‐dST1)* compared to control strain *tubGal80* ^*TS*^/*UAS‐GFP;TubGal4*/+ and the parental control *tubGal80* ^*TS*^ *;TubGal4*. Log‐rank (Mantel–Cox) test, ***P < 0.0005, n.s., not significant.HClimbing performance index of *tubGal80* ^*TS*^/*UAS‐GFP;TubGal4*/+ and *tubGal80* ^*TS*^/+*;TubGal4*/*UAS‐dST1* at different time points: 40 days (*n* = 176 and 196, respectively; biological replicates), 60 days (*n* = 159 and 178, respectively; biological replicates), and 80 days (*n* = 119 and 158, respectively; biological replicates). Upregulation of dST1 has been induced only during larval stages. Data are indicated as mean + SEM. Two‐way ANOVA, ***P < 0.0005.IClimbing performance index of male *tubGal80* ^*TS*^/*UAS‐GFP;TubGal4*/+ and *tubGal80* ^*TS*^/+*;TubGal4*/*UAS‐dST1* at different time points: 40 days (*n* = 93 and 93, respectively; biological replicates), 60 days (*n* = 79 and 81, respectively; biological replicates), and 80 days (*n* = 41 and 70, respectively; biological replicates). Upregulation of dST1 has been induced only during larval stages. Data are indicated as mean + SEM. Two‐way ANOVA, ***P < 0.0005.JClimbing performance index of female *tubGal80* ^*TS*^/*UAS‐GFP;TubGal4*/+ and *tubGal80* ^*TS*^/+*;TubGal4*/*UAS‐dST1* at different time points: 40 days (*n* = 83 and 103, respectively; biological replicates), 60 days (*n* = 80 and 97, respectively; biological replicates), and 80 days (*n* = 78 and 88, respectively; biological replicates). Upregulation of dST1 has been induced only during larval stages. Data are indicated as mean + SEM. Two‐way ANOVA; **P < 0.005, ***P < 0.0005.

As previously described, the RNA‐seq experiment performed on mouse liver (Fig 4A and B) suggested that sulfotransferases could be involved in lifespan extension induced by the rapamycin treatment. *Drosophila* harbors four *SULT* orthologues, namely *dST1*, *dST2*, *dST3*, and *dST4*, thought to be derived from gene duplication processes occurred in a common ancestral gene. Among them, *dST1* and *dST3* show a high degree of homology (Hattori *et al*, 2008; Fahmy & Baumgartner, 2013), and as shown in Fig EV5D and E, we observed increased *dST1* and *dST3* mRNA levels upon 12 h of rapamycin treatment during larval development, but not in adult (10 days old) rapamycin‐treated flies, thus supporting the idea that rapamycin has different effects based on age of administration, as observed in mammals. Of notice, dST1 (CG5428) shares 46 and 47% of similarity in the amino acid sequence with the mouse Sult2a3 and Sult2a6, respectively, that are upregulated in both male and female P4‐P30 rapamycin‐treated mice (Fig 4B). To investigate the role of *dST1* in lifespan extension, we took advantage of the GAL4‐UAS system (Brand & Perrimon, 1993) to induce *dST1* constitutive overexpression during the entire life of flies, using a *TubGal4* promoter (Fig 7A upper timeline). Lifespan was compared with a transgenic strain carrying the same genetic weight (*UAS‐GFP;tubGal4*/+). Constitutive *dST1* upregulation in transgenic flies determines a decrease in lifespan compared with control flies of both sexes (Fig 7B–D). Since prolonged upregulation seems to be detrimental, we aimed to recreate the same experimental conditions of rapamycin treatment by transiently overexpressing *dST1* during larval development only (Fig 7A lower timeline). Transient *dST1* upregulation was achieved using a temperature‐sensitive *tubGal80* ^*TS*^ *;TubGal4* strain, and the lifespan was compared with a transgenic strain carrying the same genetic weight (*tubGal80* ^*TS*^/*UAS‐GFP;tubGal4*/+). Since temperature plays a central role in lifespan, we compared the effect of *dST1* overexpression during larval stage with a control that has been raised at the same temperature/following the same temperature shifts. Therefore, the effect of the temperature on lifespan is accounted for in both the controls and the *dST1*‐overexpressing flies (Fig 7). *dST1*‐overexpressing flies displayed an extension of lifespan compared with controls (Fig 7E and Dataset EV6), and the effect was present in both males and females (Fig 7F and G and Dataset EV6). However, the effect in females was less strong probably due to a sex‐specific role of dST1. This is supported by the observation that in the male mouse livers, more sulfotransferases were dysregulated showing an higher expression level compared with females upon rapamycin treatment. These data support the previous rapamycin experiments on mice and *Drosophila* (Figs 1A and 6A) and confirmed the presence of precise time windows during which lifespan can be affected. To characterize the effect of transient *dST1* overexpression on the fitness of the flies, we tested their endurance by analyzing the motor function during aging with a negative geotaxis assay. Indeed, locomotor behavior is considered as a marker of organismal health that declines with age (Bjedov *et al*, 2010; Stefana *et al*, 2017). Early and transient *dST1* upregulation in larvae improved the climbing performance over time compared with controls (Fig 7H), and the amelioration was present in both sexes (Fig 7I and J). Overall, our novel findings suggest that lifespan can be determined during early life, unveiling the putative role of *dST1* as a new lifespan modulator.

## Discussion

Environmental and genetic components influence lifespan by regulating specific signaling pathways, metabolism, and transcription factors. So far, the great majority of studies were based on pharmacological treatments administered late in life for a prolonged time or repeated treatments. On the contrary, only a few groups studied how perturbation in the early life of mice and flies (i.e., caloric restriction/modulation or antioxidant treatments) affects lifespan (Ozanne & Hales, 2004; English & Uller, 2016; Stefana *et al*, 2017). Our results indicate a critical early‐life time frame during which the modulation of age‐related pathways determines a long‐term effect on lifespan. By exploiting a transient rapamycin treatment, we identified a crucial lifespan‐extending time window both in mouse (P4‐P30) and in *Drosophila melanogaster* (larval stage and early adult). Interestingly, the transient inhibition of the mTOR pathway in later periods of life does not significantly improve the lifespan. Our results suggest the existence of a “memory” mechanism that increases lifespan and that can be modulated in early life only. A similar hypothesis has been postulated in mice exposed to caloric restriction in adult life (Hahn *et al*, 2019). These mice showed signs of a “nutritional memory” and metabolic remodeling of white adipose tissue, but the molecular mechanisms beyond these effects are unknown (Hahn *et al*, 2019). To identify genes that affect lifespan, we analyzed gene expression profiles of livers from mice transiently treated with rapamycin early in life (P4‐P30). We chose to profile the liver, because this tissue regulates glucose, insulin signaling, and lipid homeostasis and could potentially regulate mammalian lifespan (Sengupta *et al*, 2010; Lamming & Sabatini, 2013). Furthermore, hepatic gene signatures of different lifespan‐extending treatments have been already used to identify aging‐related candidate genes (Tyshkovskiy *et al*, 2019). Indeed, P4‐P30 rapamycin‐treated mice processed on the last day of treatment show a unique and distinct hepatic gene signature, different from the other transient treatments. Of notice, this signature is not maintained during later time points. Indeed, gene expression changes cannot be observed in middle life (P350). These results led us to speculate that the P4‐P30 modulation of the mTOR pathway determines a chain of events set in motion during the early‐life time window, revealing new age‐related genes with novel functions in the regulation of lifespan. Interestingly, although the analysis showed a sex difference in the transcriptome changes (Fig 3), no sex difference in lifespan extension was observed in response to rapamycin treatments (Fig 1). We can speculate that the similar increase in mouse lifespan (in the two sexes) might be achieved through both transcriptional and translation regulation.

Of notice, a preprint project by Gladyshev and collaborators supports and strengthens our data, independently discovering a similar time‐dependent effect by administering rapamycin transiently in early life (preprint: Shindyapina *et al*, 2022). Importantly, GSEA on P4‐P30 rapamycin‐treated mice (males) resulted in the enrichment of several molecular functions related to sulfotransferase activity (steroid, bile salt, and alcohol sulfotransferase activity), while *Sult2a3* and *Sult2a6* were found to be upregulated in both sexes. *SULTs* are generally involved in the xeno‐ and endobiotic metabolism that is divided into three phases: (I) modification, (II) conjugation, and (III) excretion. Those enzymes catalyze the transfer of sulfonate (SO_3_^−^) to a hydroxyl or amino group, favoring the elimination from the body (Gamage *et al*, 2006). Sulfonation occurs on numerous xeno‐ and endobiotics such as drugs, steroid hormones, bile acids, peptides, and lipids, and it has been generally considered as a detoxification pathway generating an end product that is more amenable to eliminate (Gamage *et al*, 2006). Xenobiotic metabolism has been already associated with lifespan extension in *Drosophila melanogaster*. Indeed, early low doses of oxidants determine a long‐term mechanism that leads to lifespan extension (Obata *et al*, 2018). However, the constitutive upregulation of xenobiotic resistance mediators correlates with health span amelioration, but not with lifespan extension (Afschar *et al*, 2016). For this reason, we decided to test the early‐transient lifespan‐extension role of sulfotransferase taking advantage of *Drosophila melanogaster* that has a considerably shorter lifespan than mice and it is characterized by the presence of genetic tools that allow temporal modulation of gene expression. Indeed, transient *dST1* overexpression (*Sult2a3* and *Sult2a6 Drosophila* homologs) during larval development determines a small but significant increase in lifespan revealing a new putative function of *dST1* as an early regulator of the aging process. Our findings indicate a link between early‐life events and long‐term effects on lifespan indicating the existence of a critical time window that can permanently affect how long an individual can live. We found that the modulation of gene expression/pathways in this specific time window can determine lifespan extension both in mice and in *Drosophila melanogaster*. Further studies are needed to assess the role of sulfotransferases and their regulators in the aging process and to unveil new drugs that might increase lifespan through an early and transient administration. However, our data represent a new starting point for the study of lifespan, paving the path for future work on humans.

## Materials and Methods

### Mice

CD1 mice (cat. #022) were purchased from Charles River and housed in a certified specific pathogen‐free (SPF) Animal Facility in accordance with European Guidelines. Mice were provided ad libitum food access throughout their lifetime. Male and female CD1 mice were treated with rapamycin or ethanol at different time points and used for the survival analyses. Mice were monitored daily until human endpoint, caused by death or euthanasia due to the occurrence of severe age‐related pathologies, identified by veterinary and biological services staff members. Survival analysis was performed using mice born within the same period (3–4 months), and all of them derived from a small cohort of male and female CD1 mice, thus decreasing the differences in the genetic background The experiments were approved by the Italian Ministry of Health as conforming to the relevant regulatory standards “Tutela del benessere animale, igiene zootecnica e igiene urbana veterinaria”.

### Rapamycin treatment and survival analysis in mice

Rapamycin (Alfa Aesar, cat. #J62473) was dissolved in ethanol at 20 mg/ml and then diluted in milli‐Q water. CD1 mice were daily intraperitoneally injected with rapamycin (10 mg/kg) or ethanol for two distinct time windows from P4 to P30 or from P30 to P60 and sacrificed at the end of treatments for RNA‐seq analysis or monitored until human endpoint for the survival analysis. The acute rapamycin effect was evaluated by intraperitoneally injecting CD1 mice with either rapamycin (10 mg/kg) or ethanol from P4 to P8 or from P30 to P34 and sacrificed at the end of treatments for Western blot and RNA‐seq analyses. Control and rapamycin‐treated animals were defined per cage to ensure similar mother feeding among the rapamycin‐treated mice. Control and rapamycin‐treated mice were weaned at the same age.

### Weight analysis

Rapamycin‐treated and control CD1 mice were weighed daily in the morning to check weight changes during and after rapamycin treatment. Weight measurements were collected from all the different time windows: P4‐P30 control (*n* = 3), P4‐P30 rapamycin (*n* = 2), P30‐P60 control (*n* = 3), and P30‐P60 rapamycin (*n* = 3). Mean weight measurements of each litter were calculated considering both males and females.

### Length analysis

Measurements have been performed on P4‐P30 and P30‐P60 rapamycin‐treated mice at 15 and 20 months. Mean length measurements were calculated considering both males and females.

### Western blot

Mouse section: Whole livers of mice subjected to 4 days of treatment (P4‐P8 and P30‐P34) were dissected and snap‐frozen from male and female CD1 mice at P8 and P34, respectively. Fresh‐frozen livers were smashed with mortar and pestle, and proteins were extracted from smashed tissues in lysis buffer (50 mM Tris–HCl, 150 mM NaCl, 20 mM EDTA, 1% NP‐40, 0.5% sodium deoxycholate, and 0.1% SDS), supplemented with proteases inhibitors (VWR, cat. #M221‐1ML), DTT (Thermo Fisher Scientific, cat. #R0861), and SERVA Electrophoresis™ Phosphatase Inhibitor Mix II Solution (Thermo Fisher Scientific, cat. #3905501). *Drosophila* section: L3 wandering larvae (*n* = 30) and 10‐day‐old flies (*n* = 10) treated for 3 days with rapamycin or ethanol, was collected and homogenized using a pellet pestle (Sigma‐Aldrich, cat. #Z359971‐1EA) to obtain a better homogenization of the larva and adult fly whole bodies in the same lysis buffer used for mouse whole‐liver extracts (see above). Samples in lysis buffer were left in ice for 30 min and then centrifuged at 18,000 *g* for 20 min. Supernatants, containing extracted proteins, were collected in new Eppendorf tubes, and proteins were quantified using the Bradford method and stored at −80°C. Proteins were resolved by SDS–PAGE and transferred onto a PVDF membrane (pore size 0.2 μm, Merck, cat. #GE10600021). The membrane was blocked in 5% BSA (Thermo Fisher Scientific, cat. #11423164)/TBS‐T (0.1% Tween in TBS) for 1 h at room temperature and low agitation, and subsequently probed with primary antibodies, diluted in 5% BSA/TBS‐T, overnight at 4°C. Then, the membrane was washed with TBS‐T three times for 10 min and incubated with secondary antibodies, diluted in 5% BSA/TBS‐T, for 1 h at room temperature. After another cycle of three washes with TBS‐T, protein levels were detected using the Clarity Western ECL Substrate (Bio‐Rad, cat. #1705062). The harsh stripping protocol was applied to detect the phosphorylation state of protein in the same PVDF membrane. To remove primary and secondary antibodies, the membrane was incubated with a stripping buffer (20 ml 10% SDS, 12.5 ml 0.5 M Tris–HCl, pH 6.8, 67.5 ml distilled water supplemented with 0.8 ml β‐mercaptoethanol (Scharlab, cat. # ME00950250)) at 50°C for 45 min. Then, the membrane was rinsed with milli‐Q water for 1–2 min and with TBS‐T for 5 min. After another step of blocking, the membrane was incubated with a new primary antibody.

### RNA extraction, library generation and sequencing

P4‐P8 (*n* = 3), P4‐P30 (*n* = 5), P30‐P34 (*n* = 3), P30‐P60 (*n* = 3), and P350 (*n* = 3) control and rapamycin‐treated whole livers were dissected and snap‐frozen from male and female CD1 mice at P8, P30, P34, P60, and P350, respectively. Fresh‐frozen livers were smashed with mortar and pestle, and total RNA was isolated from smashed tissues with TRIzol Reagent (Invitrogen, cat. #15596018), according to the manufacturer's instructions. Pellet pestles (Sigma‐Aldrich, cat. #Z359971‐1EA) were used to obtain a better homogenization of the hepatic tissue in TRIzol. Then, RNA quality was controlled with the High Sensitivity RNA Assay at the 2100 Bioanalyzer (Agilent, cat. #G2939BA) and the extracted RNA was stored at −80°C until the RNA‐seq analysis. Libraries were prepared from the extracted RNAs using the QuantSeq 3′mRNA‐Seq Library Prep Kit FWD (Cat. No. LX01596 Lexogen, Vienna, Austria) using 1 μg of RNA per library and following the manufacturer’s instructions. We modified the standard protocol by adding Unique Molecular Identifiers (UMI) during the second strand synthesis step. Indices from the Lexogen i7 6nt Index Set and i5 6nt Dual Indexing Add‐on Kits (Cat. No. 044.96 and 047.96, Lexogen) were used, and 15 cycles of library amplification were performed. Libraries were eluted in 30 μl of the kit's Elution Buffer. The double‐stranded DNA concentration was quantified using the Qubit dsDNA HS Assay Kit (Thermo Fisher), ranging from 3 to 12 ng/μl. The molar concentration of cDNA molecules in the individual libraries was calculated from the double‐stranded DNA concentration and the single library average size (determined on a PerkinElmer LabChip GX). An equal number of cDNA molecules from each library were pooled, and the final pool was purified once more in order to remove any free primer and prevent index‐hopping. The pooled libraries were sequenced in a NovaSeq 6000 instrument (Illumina, San Diego, CA) on an SP flowcell, producing 900 M single reads of 100 nt.

Sequencing reads from the resulting FASTQ files were aligned onto mouse reference genome (GRCm38 primary genome assembly) using STAR aligner version 2.7.7a (Dobin *et al*, 2013), setting the parameters outFilterScoreMinOverLread and outFilterMatchNminOVerLread to the value 0.33. The resulting SAM files were sorted and converted to BAM files using SAMtools (Li *et al*, 2009). Transcript counts were computed using the featureCounts function available from the Rsubread R package (Liao *et al*, 2019), utilizing mouse gene annotation (GRCm38) for read summarization. 6‐ and 12‐month‐old adult chronic rapamycin treatment RNA‐seq data counts were downloaded from Gene Expression Omnibus (GEO) under accession number GSE131754 (Tyshkovskiy *et al*, 2019). Transcripts with a raw count lower than 20 in all biological replicates across the considered conditions were excluded. TMM (trimmed mean of M values) normalization and CPM (counts per million) conversion were performed to obtain normalized transcript levels.

### Differential expression analysis

The edgeR R package (Robinson *et al*, 2010) was used to perform differential expression analysis. Rapamycin‐treated samples were compared against the respective control samples. Transcripts with a log2 fold change higher/smaller than 1/−1, an FDR‐corrected *P* < 0.05 and a mean log2 CPM > 0 across the replicates of at least one of the two compared groups were considered as significantly differentially expressed genes (DEGs). To account for potential effects due to biological replicates generated in different sequencing batches, differential expression analysis of P4‐P30 male and female mice was performed considering the batch as a covariate in the analysis model.

### Gene set enrichment analysis

Gene Set Enrichment Analysis (GSEA) of Gene Ontology (GO), biological process (BP), molecular function (MF), and cellular component (CC) terms was performed with the gseGO function of the clusterProfiler R package (Yu *et al*, 2012), and *P*‐values were FDR‐corrected.

As described in Tyshkovskiy *et al*, 2019, the input for the gseGO function was obtained by preranking the list of genes of the edgeR output based on the −log10(adjusted *P*‐value) corrected by the sign of regulation (1, −1, or 0 if the log2FC value is positive, negative, or equal to 0, respectively), as:

$$-\log10\left(\mathrm{adj}\;p\;\text{value}\right)*\mathrm{sign}\left(\log2\mathrm{FC}\right)$$

Significance scores of enriched functions were obtained from the output of the gseGO function based on −log10(q‐values) corrected by the sign of the normalized enrichment score (NES), as:

$$\text{significance score}=-\log10\left(q\;\text{values}\right)*\mathrm{sign}\left(\mathrm{NES}\right)$$

Significance score barplots were inspected manually to choose for terms that are statistically significant in the P4‐P30 male or female categories.

### Metabolomics on mouse livers

Metabolomic data were obtained by liquid chromatography coupled to tandem mass spectrometry. We used an API‐3500 triple quadrupole mass spectrometer (AB Sciex, Framingham, MA, USA) coupled with an ExionLC™️ AC System (AB Sciex). Cells were smashed in a Tissue Lyser for 2 min at maximum speed in 250 μl of ice‐cold methanol/water/acetonitrile 55:25:20 containing [U‐^13^C_6_]‐glucose 1 ng/μl and [U‐^13^C_5_]‐glutamine 1 ng/μl as internal standards. Lysates were spun at 15,000 *g* for 15 min at 4°C, and supernatants were then passed through a regenerated cellulose filter (4 mm Ø, Sartorius). Samples were then dried under N2 flow at 40°C and resuspended in 125 μl of ice‐cold methanol/water 70:30 for subsequent analyses.

The quantification of amino acids, their derivatives, and biogenic amine was performed through previous derivatization. Briefly, 25 μl of 125 μl samples was collected and dried separately under N2 flow at 40°C. Dried samples were resuspended in 50 μl of phenyl‐isothiocyanate (PITC), EtOH, pyridine and water (5%:31.5%:31.5%:31.5%) and then incubated for 20 min at RT, dried under N2 flow at 40°C for 90 min, and finally resuspended in 100 μl of 5 mM ammonium acetate in MeOH/H2O 50:50. Quantification of different amino acids was performed using a C18 column (Biocrates, Innsbruck, Austria) maintained at 50°C. The mobile phases for positive ion mode analysis were phase A: 0.2% formic acid in water and phase B: 0.2% formic acid in acetonitrile. The gradient was T0: 100% A; T5.5: 5% A; and T7: 100% A with a flow rate of 500 μl/min. All metabolites analyzed in the described protocols were previously validated by pure standards, and internal standards were used to check instrument sensitivity.

Quantification of energy metabolites, cofactors, and nucleotides was performed using a cyano‐phase LUNA column (50 mm × 4.6 mm, 5 μm; Phenomenex) by a 5‐min run in negative ion mode with two separated runs: *Protocol A*—mobile phase A was water and phase B was 2 mM ammonium acetate in MeOH, and the gradient was 10% A and 90% B for all the analysis with a flow rate of 500 μl/min; and *Protocol B*—mobile phase A was water and phase B was 2 mM ammonium acetate in MeOH, and the gradient was 50% A and 50% B for all the analysis with a flow rate of 500 μl/min.

The quantification of acylcarnitines, GSH, GSSG and SAMe was performed on the same samples using a Varian Pursuit XRs Ultra 2.8 Diphenyl column (Agilent). Samples were analyzed by a 9‐min run in a positive ion mode. Mobile phases were A: 0.1% formic acid in H20 and B: 0.1% formic acid in MeOH, and the gradient was T0: 35% A; T2.0: 35% A; T5.0: 5% A; T5.5: 5% A; T5.51: 35% A; and T9.0: 35% A with a flow rate of 300 μl/min.

Bile acids were analyzed on an API‐4000 triple quadrupole mass spectrometer (AB Sciex) coupled with a HPLC system (Agilent) and CTC PAL HTS autosampler (PAL System). The LC mobile phases were (A) 10 mM NH_4_Ac and 0.015% formic acid in water, and (B) 10 mM NH_4_Ac and 0.015% formic acid in acetonitrile/methanol/water (65/30/5). The gradient was as follows: T0: 65% A (flow rate 400 μl/min); T0.7: 60% A (flow rate 400 μl/min); T3: 55% A (flow rate 400 μl/min); T3.2: 45% A (flow rate 400 μl/min); T5.5: 35% A (flow rate 450 μl/min); T6.5: 0% A (flow rate 500 μl/min); T8.5: 0% A (flow rate 600 μl/min); T8.6: 65% A (flow rate 700 μl/min); and T11: 65% A (flow rate 400 μl/min). The Hypersil GOLD column C18 (100 mm × 3 mm, 3 μm) was maintained at 50°C. The mass spectrometer was operated in negative ion mode and in selective ion monitoring SIM/SIM mode.

MultiQuant™ software (version 3.0.3; AB Sciex) was used for data analysis and peak review of chromatograms.

Raw areas were normalized by the median of all metabolite areas in the same sample. Specifically, we defined the relative metabolite abundance $m_{a}^{N}$ as:

$$m_{a}^{N}=\frac{\mathrm{Xn}}{M_{a=1}^{n}a}$$

where *Xn* represents the peak area of metabolite *n* for samples *a*, *b*, …, *z*, and $M_{a=1}^{n}a$ represents the median of peak areas of metabolite *n* for samples *a*, *b*, *…*, *z*. Obtained data were then transformed by log10 transformation and Pareto‐scaled to correct for heteroskedasticity, reduce the skewness of the data, and reduce mask effects (Ghaffari *et al*, 2019). In detail, obtained values were transformed by log10 and then scaled by Pareto's method as follows:

$${\bar{X}}_{\mathrm{ij}}=\frac{x_{\mathrm{ij}}-{\bar{x}}_{i}}{\sqrt{s_{i}}}$$

where *x*_*ij*_ is the transformed value in the data matrix (*i* (metabolites), *j* (samples)), and *s*_*i*_ is the standard deviation of transformed metabolite values (van den Berg *et al*, 2006). Obtained values were considered as relative metabolite levels. Data processing and analysis were performed by the MetaboAnalyst 5.0 web tool (Chong *et al*, 2019).

### Frailty index assessment

Frailty Index assessment was performed at 7, 15, and 20 months on P4‐P30 and P30‐P60 rapamycin‐treated mice. The clinical FI score for each mouse was calculated using a modified version of the tool published previously by Whitehead *et al* (2014). Briefly, mice were placed in a fresh cage and moved to a dedicated small animal procedure room designed for behavioral testing. Mice were weighed, and then, a series of noninvasive observations on 30 clinical items were taken (as listed in Dataset EV1). Clinical assessment included evaluation of the integument, musculoskeletal system, vestibulocochlear and auditory systems, ocular and nasal systems, digestive system, urogenital system, respiratory system, signs of discomfort, and the body weight (grams). A complete list of the clinical signs of deterioration and/or deficits evaluated in this study can be found in Dataset EV1.

### Forelimb grip strength

A grip strength meter system (Cat. No. 47200, Ugo Basile Srl) was used to assess grip strength at 7, 15 and 20 months on P4‐P30 and P30‐P60 rapamycin‐treated mice. Mice were allowed to hold onto the grid with the front paws. Each mouse was given 3 trials over the course of 5 min. Average values (gF) were then normalized using the weight (g) to calculate the grip strength of an individual mouse.

### *Drosophila* stocks

*Drosophila* strains were raised on standard cornmeal and molasses medium in fly food vials (25 x 95 mm, Biosigma, cat. #789008) at 25°C where not otherwise indicated. Lyophilized Nutri‐Fly food (Genesee Scientific, cat. #789211) was used during the rapamycin treatment window. The stocks used in this study were as follows: *white iso31 (w* ^*iso31*^ in the text, kind gift from Alex Gould), y[1] w[*]; P{w[+*mC] = UAS‐mCD8::GFP*.*L}LL5*, *P{UAS‐mCD8::GFP*.*L}2* (BDSC_5137‐*UAS‐GFP* in the text), *P{w[*+*mC] = tubP‐GAL80[ts]}10; P{w[*+*mC] = tubP‐GAL4}LL7*/*TM6B*, *Tb[1]* (BDSC_86328‐*TubGal80;TubGal4* in the text), y[1] w[*]; P{w[+*mC] = tubP‐GAL4}LL7*/*TM3*, *Sb[1] Ser[1]* (BDSC_5138‐TubGal4 in the text) purchased from Bloomington *Drosophila* Stock Center and *M{UAS‐St1*.*ORF*.*3xHA*.*GW}ZH‐86Fb* (cat. #F003139, *UAS‐dST1* in the text) purchased from FlyORF.

### Rapamycin treatment and survival analysis in *Drosophila*

#### Larval transient treatment

Rapamycin (Alfa Aesar, cat. #J62473) was dissolved in ethanol at a final concentration of 20 mg/ml, and then, working aliquots were diluted in milli‐Q water up to a concentration of 1, 50, and 200 μM. For each experiment of survival analysis, three independent cohorts of *w* ^*iso31*^ flies were established at 25°C on Nutri‐Fly food, to favor the distribution of rapamycin within the food. Adult flies were allowed to lay eggs in the test tubes for 12 h leading to egg synchronization. 72 h after egg laying, 1 ml of 1, 50, or 200 μM rapamycin solutions or ethanol as control was added drop by drop in each tube. Newly eclosed flies were promptly collected, divided by sex, and maintained in new vials containing standard food at a density of 10 ± 3 flies per vial. Flies were flipped to new vials at least twice a week and monitored for death events.

#### Adult early‐transient treatment

Five cohorts of *w* ^*iso31*^ flies were established on standard food. Adult flies were allowed to lay eggs in the test tubes for 12 h leading to egg synchronization. Newly eclosed flies were collected, divided by sex, and maintained in new vials containing standard food at a density of 10 ± 3 flies per vial. *From days 0 to 10*: On the first day post‐eclosion, flies were transferred to new vials containing Nutri‐Fly food supplemented with 1 ml of ethanol or 200 μM of rapamycin. Flies were maintained in the Nutri‐Fly food for 10 days (flipped three times) and then transferred back to fresh vials with standard food. *From days 10 to 13*: On the tenth day post‐eclosion, flies were transferred to new vials containing Nutri‐Fly food supplemented with 1 ml of ethanol or 200 μM of rapamycin. Flies were maintained in the Nutri‐Fly food for 3 days and then transferred back to fresh vials with standard food. *From days 10 to 20*: On the tenth day post‐eclosion, flies were transferred to new vials containing Nutri‐Fly food supplemented with 1 ml of ethanol or 200 μM of rapamycin. Flies were maintained in the Nutri‐Fly food for 10 days (flipped three times a week) and then transferred back to fresh vials with standard food. For all the time points listed above, flies were flipped to new vials at least twice a week and monitored for death events.

### *Drosophila’*s food receipts

Flies were reared on standard sugar/yeast/agar food (standard food) or Nutri‐Fly food, based on the experimental setup. Standard food consists of 85 g corn flour, 60 g sugar, 30 g brewer's yeast (ACROS Organics™, cat. # 368080050), 10 g Insectagar ZN5 (B.&V. S.R.L. The Agar Company), 50 g Molasses, 15 ml/l 1 M Propionic Acid (Genesee Scientific, cat. #789177), and 15 ml/l 10% w/v Tegosept in EtOH 96% (Genesee Scientific, cat. #789063) per liter.

Lyophilized food consists of 200 g Nutri‐Fly food (Genesee Scientific, cat. #789211) and 16.25 g brewer's yeast (ACROS Organics™, cat. # 368080050) per liter.

### Transient overexpression of dST1

#### Larval transient overexpression

To study the effect of transient dST1 overexpression on *Drosophila* lifespan, the following crosses were established on standard food: *;TubulinGal80* ^*ts*^ *;TubulinGal4*/*TM6B* flies with *;;UAS‐dST1 flies*, *;TubulinGal80* ^*ts*^ *;TubulinGal4*/*TM6B* flies with *;UAS‐GFP*/*Cyo;* flies and *;TubulinGal80* ^*ts*^ *;TubulinGal4*/*TM6B* flies with *;TubulinGal80* ^*ts*^ *;TubulinGal4*/*TM6B* flies. Adult flies, maintained at 18°C, were allowed to lay eggs in the test tubes for 12 h leading to egg synchronization. 130 h after egg laying, vials containing developing larvae were shifted to 29°C, to induce the temperature‐sensitive inhibition of the Gal80 and allow the overexpression of the genes of interest (dST1 or GFP) until puparium formation, and then maintained at 18°C until death. Adult flies of each cross were selected for the following genotypes: *;TubulinGal80* ^*ts*^/+*;TubulinGal4*/*UAS‐dST1* (in the text *TubGal80;TubGal4;UAS‐dST1*), *;TubulinGal80* ^*ts*^/*UAS‐GFP;TubulinGal4*/+ (in the text *TubGal80;TubGal4;UAS‐GFP*) and *;TubulinGal80* ^*ts*^ *;TubulinGal4* (in the text *TubGal80;TubGal4*). Selected flies were divided by sex in new vials containing standard food, at a density of 10 ± 3 flies per vial. Flies were flipped at least twice a week and monitored for death events.

### Constitutive dST1 overexpression

To study the effect of constitutive dST1 overexpression on *Drosophila* lifespan, the following crosses were established on standard food: *;;TubGal4*/*TM6B flies with ;;UASdST1 flies*, *;;TubGal4*/*TM6B flies with ;UAS‐GFP*/*Cyo;* flies. Adults were allowed to lay eggs in the test tubes for 12 h leading to egg synchronization. Male and female flies were selected after eclosion based on the specific genotype for the cross (*;;TubGal4;UAS‐dST1*, *;;TubGal4;UAS‐GFP*), and maintained at 25°C in new vials. Flies were flipped three times a week and monitored for death events.

### Antibodies

Primary antibodies used are listed here: Rabbit anti‐Phospho‐S6 Ribosomal Protein (Ser235/236) (1:1,000, Cell Signaling, cat. #2211), Rabbit S6 Ribosomal Protein (5G10) (1:4,000, Cell Signaling, cat. #2217), anti‐dS6K (generous gift from Aurelio Teleman 1:3,000), and anti‐phospho‐Thr398‐S6K (Cell Signaling Technologies #9209, 1:1,000). The secondary antibodies used are Peroxidase AffiniPure Goat Anti‐Rabbit IgG (H+L) (1:5,000, Jackson ImmunoResearch, cat. #111‐035‐003) and Rabbit anti‐Guinea Pig IgG (H+L) (1:5,000 Thermo Fisher, cat.# HRP 61‐4620).

### RNA isolation and real‐time PCR analysis

*Drosophila*: Three biological replicates of L3 wandering larvae (*n* = 30) and 10‐day‐old flies (*n* = 10) treated for 12 h with rapamycin or ethanol were snap‐frozen and stored at −80°C. Total RNA from frozen samples was isolated with TRIzol Reagent (Invitrogen), using pellet pestles (Sigma‐Aldrich, cat. #Z359971‐1EA). RNA was reverse‐transcribed using iScript cDNA synthesis kit (Bio‐Rad, cat. #1708891) according to the manufacturer's instructions, and quantitative PCR was performed using Power SYBR Green PCR Master Mix (Applied Biosystems, cat. #4367659). The Ct values were normalized to the housekeeping gene *tubulin*. Primer sequences used for qRT–PCR are listed in Table EV1.

### Climbing performance assay

Adult flies were placed in a plastic cylinder. The cylinder was tapped quickly, and it was recorded the number of flies over 5 centimeters after 15 s. This step was repeated three times.

### Statistical analysis

Survival analysis was performed calculating the lifespan in days for every mouse or *Drosophila*, and data were displayed using the Kaplan–Meier curve. The statistical significance of the results was tested using the log‐rank (Mantel–Cox) test.

The data of weight and length analyses are presented as mean + SD of three litters. Two‐tailed Student's *t*‐test was used for calculating significance values between treated and control mice.

For qRT–PCR analysis, data are presented as mean + s.e.m. of three biologically independent samples and a two‐tailed Student's *t*‐test was used to calculate significance.

For forelimb grip strength and FI analyses, data are presented as mean + s.e.m. and a two‐way ANOVA was used to calculate significance.

## Data availability

The datasets produced in this study are available in the following databases:

Metabolomic data of the liver of P4‐P30 and P30‐P60 male and female mice can be found at the private link: https://figshare.com/s/7c5f3b1ef26a2f7d5e9c

RNA‐seq experiments from liver of control and rapamycin‐treated mouse BAM files have been deposited at BioProject under the accession number PRJNA693135 (https://www.ncbi.nlm.nih.gov/bioproject/?term=PRJNA693135).

## Author contributions

**Giuseppe Aiello:** Conceptualization; methodology. **Cosimo Sabino:** Conceptualization; methodology. **Francesco Antonica:** Conceptualization. **Alessandro Quattrone:** Supervision. **Nico Mitro:** Conceptualization; supervision. **Alessandro Romanel:** Conceptualization; supervision; methodology. **Alessia Soldano:** Conceptualization; supervision; methodology. **Luca Tiberi:** Conceptualization; supervision; methodology.

In addition to the CRediT author contributions listed above, the contributions in detail are:

GA and LT designed the study, analyzed the data, and wrote the manuscript. AS supervised the *Drosophila melanogaster* experiments. FA, AS, and AQ helped in manuscript revision. GA with help from CS, MG, CB, and AS performed all the experiments. GA and CS performed *in vivo* experiments. DP and AR performed bioinformatics analyses. MA and NM performed metabolomic data and analyses.

## Disclosure and competing interests statement

The authors declare that they have no conflict of interest.

## Supporting information



Expanded View Figures PDF

Table EV1

Dataset EV1

Dataset EV2

Dataset EV3

Dataset EV4

Dataset EV5

Dataset EV6
