# Supplementary material for: Transient rapamycin treatment during developmental stage extends lifespan in Mus musculus and Drosophila melanogaster
Source: EMBO Rep. 2022 Jul 7;23(9):e55299. doi: 10.15252/embr.202255299 (PMC9442325; doi:10.15252/embr.202255299)
Supplement: Supplementary file 1 — Expanded View Figures PDF [file EMBR-23-e55299-s003.pdf]

Expanded View Figures

Figure EV1. Analysis of the physical status of P4-P30 and P30-P60 rapamycin-treated mice at different time points.

A, B Upper panels: Schematic illustration of the experimental procedures. Rapamycin effects on body and organ size in P4-P30 rapamycin-treated mice. Representative images of liver, brain, kidney, and spleen have been taken at P30 (A) and P80 (B). Scale bar: 3 cm.

C Upper panels: Representative images showing rapamycin effects on mice body size during P30-P60 treatment. Images of $n = 10$ biological replicates (both for control and for rapamycin-treated mice) have been taken at P30, P40, and P60 (end of treatment). Scale bar: 3 cm. Lower panels: Scatter dot plot indicating body weight of control and P30-P60 rapamycin-treated mice during treatment. Data are indicated as mean + SD. Two-tailed Student's *t*-test; $^*P < 0.05$, n.s., not significant.

D Upper panels: Representative images showing rapamycin effects on mice body size after P30-P60 treatment. Images of $n = 10$ biological replicates (both for control and for rapamycin-treated mice) have been taken at P70, P90, and P150. Scale bar: 3 cm. Lower panels: Scatter dot plot indicating body weight of control and P30-P60 rapamycin-treated mice after treatment. Data are indicated as mean + SD. Two-tailed Student's *t*-test; $^*P < 0.05$.

E Dot plots indicating the total length (mm) of P4-P30 and P30-P60 rapamycin-treated mice. Red scatter dots indicate the measurements of P4-P30 rapamycin-treated mice at 15 months ($n = 18$) and 20 months ($n = 12$). Blue scatter dots indicate the measurements of P30-P60 rapamycin-treated mice at 15 months ($n = 9$) and 20 months ($n = 7$). Data are indicated as mean + SEM.

F Dot plots indicating the force (Newton/grams) resulted from grip strength analysis of P4-P30 and P30-P60 rapamycin-treated mice. Red scatter dots indicate the measurements from P4-P30 rapamycin-treated mice at 7 months ($n = 20$), 15 months ($n = 18$), and 20 months ($n = 12$). Blue scatter dots indicate the measurements from P30-P60 rapamycin mice at 7 months ($n = 14$), 15 months ($n = 9$), and 20 months ($n = 7$). Data are indicated as mean + SEM.

Data information: Two-way ANOVA; $^*P < 0.05$, $^{***}P < 0.0005$, n.s., not significant.

© 2022 The Authors

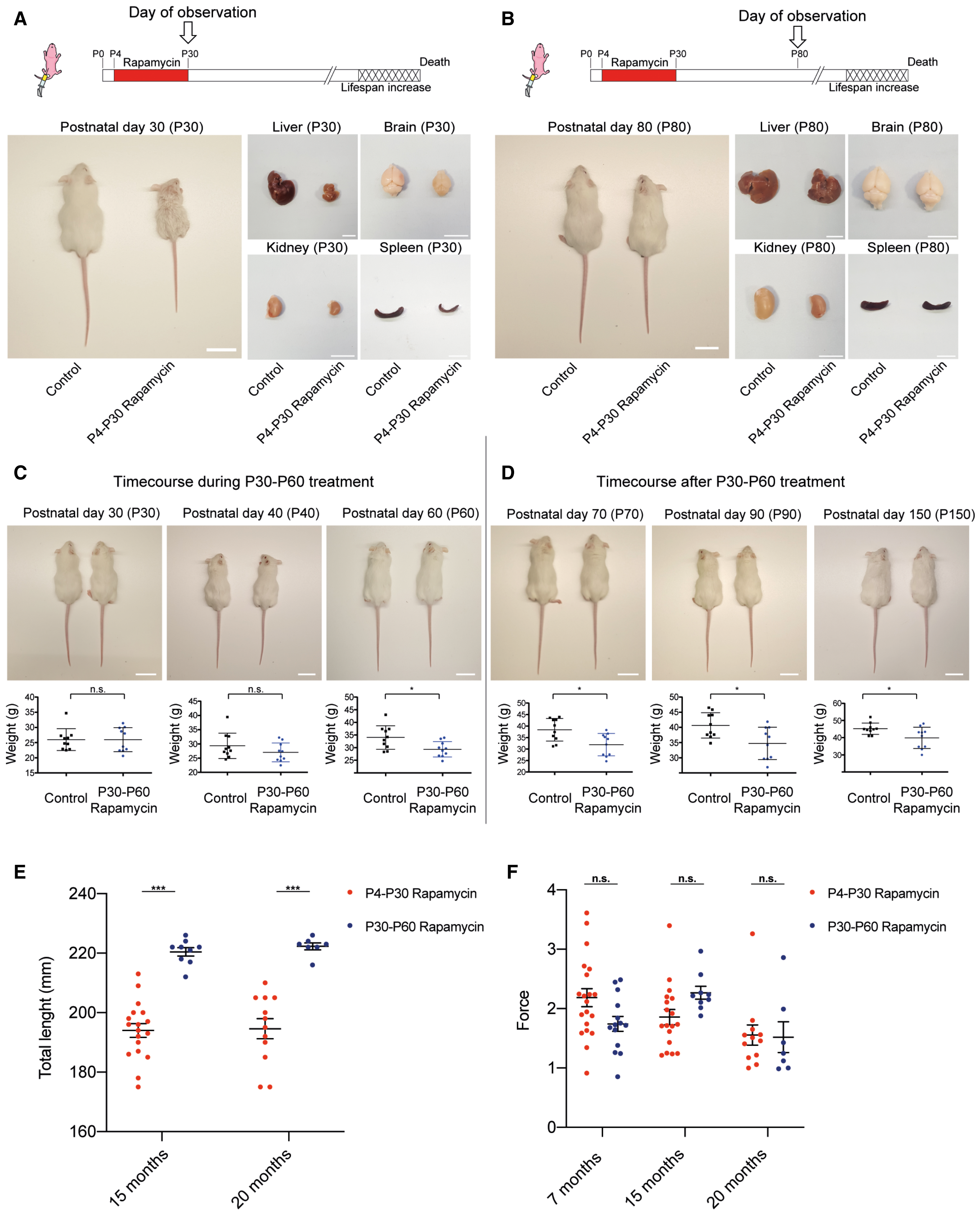
A
Day of observation
P0 P4 Rapamycin P30
Death
Lifespan increase
Postnatal day 30 (P30)
Liver (P30)
Brain (P30)
Kidney (P30)
Spleen (P30)
Control
P4-P30 Rapamycin
B
Day of observation
P0 P4 Rapamycin P30 P80
Death
Lifespan increase
Postnatal day 80 (P80)
Liver (P80)
Brain (P80)
Kidney (P80)
Spleen (P80)
C
Timecourse during P30-P60 treatment
Postnatal day 30 (P30)
Postnatal day 40 (P40)
Postnatal day 60 (P60)
D
Timecourse after P30-P60 treatment
Postnatal day 70 (P70)
Postnatal day 90 (P90)
Postnatal day 150 (P150)
Weight (g)
n.s.
*
Control
P30-P60 Rapamycin
E
Total lenght (mm)
***
P4-P30 Rapamycin
P30-P60 Rapamycin
15 months
20 months
F
Force
n.s.
7 months
15 months
20 months

Figure EV1.

Figure EV2. RNA-seq analysis of acute rapamycin treatments (P4-P8 and P30-P34).

A Gene Set Enrichment Analysis (GSEA) results of P4-P30 and P30-P60 treatments in male and female mice. Significance score, calculated as log10 (q-value) corrected by the sign of regulation, is plotted on the y-axis. The whole list of enriched GO terms is available in Dataset EV4.

B Landscape of up- and downregulated genes across the P4-P8 and P30-P34 treatments in male and female mice. Venn diagrams are used to highlight private and shared differentially expressed genes.

C Expression profile of *Nr1i3* (*CAR*) gene across P4-P8 and P30-P34 male (left side) and female (right side) rapamycin-treated mice. Values in treated and control samples across the different conditions are shown as median CPM with bars representing standard deviations across the biological replicates. Data distribution is presented through boxplots, where the central bar represents the median, while the lower and upper hinges correspond to the first and third quartiles (the 25th and 75th percentiles). The upper/lower whisker extends from the hinge to the largest/smallest value no further than 1.5 * IQR from the hinge (where IQR is the interquartile range, or distance between the first and third quartiles). *P*-values were generated by the edgeR DEG analysis, detailed in the Methods. *$P < 0.05$, ***$P < 0.0005$, n.s., not significant.

 © 2022 The Authors

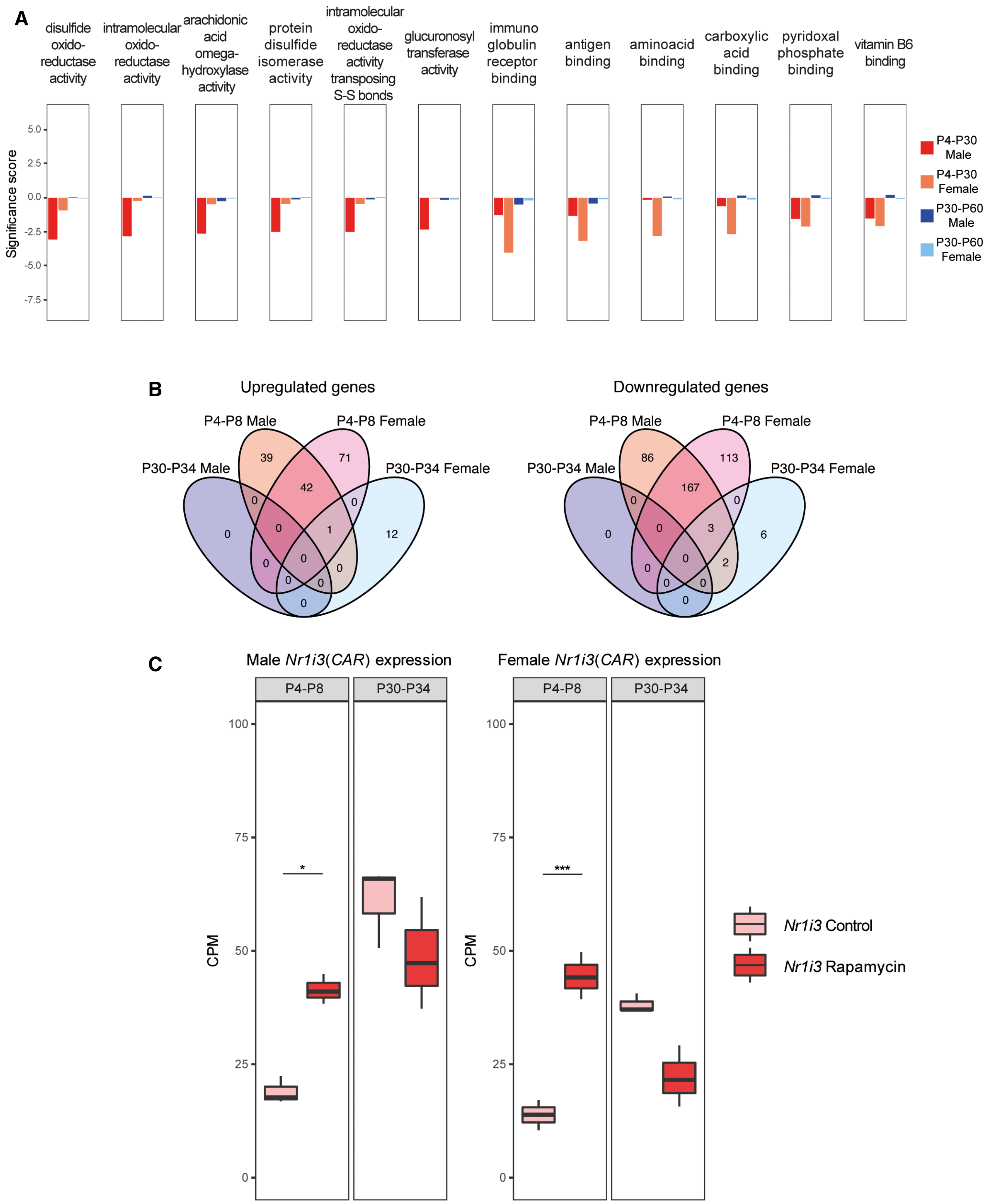
A
disulfide oxido-reductase activity
intramolecular oxido-reductase activity
arachidonic acid omega-hydroxylase activity
protein disulfide isomerase activity
intramolecular oxido-reductase activity transposing S-S bonds
glucuronosyl transferase activity
immuno globulin receptor binding
antigen binding
aminoacid binding
carboxylic acid binding
pyridoxal phosphate binding
vitamin B6 binding
Significance score
P4-P30 Male
P4-P30 Female
P30-P60 Male
P30-P60 Female
B
Upregulated genes
Downregulated genes
P4-P8 Male
P4-P8 Female
P30-P34 Male
P30-P34 Female
C
Male Nr1i3(CAR) expression
Female Nr1i3(CAR) expression
P4-P8
P30-P34
CPM
Nr1i3 Control
Nr1i3 Rapamycin

Figure EV2.

Figure EV3. Metabolic changes in early-transient control mice.

A, B Principal component analysis (PCA) (A) and heatmap (B) of liver metabolomic profile from P4-P30 (green samples) and P30-P60 (red samples) mice treated with vehicle.

C Volcano plots showing the metabolomic changes in P4-P30 compared with P30-P60 mice treated with vehicle. Each circle represents one metabolite. The log2 fold change is represented on the x-axis. The y-axis shows the -log10 of the FDR. A FDR of 0.1 is indicated by gray line. Gray, pink, and light blue dots represent unchanged, significantly downregulated, and significantly upregulated metabolites, respectively. Blue dots represent significantly upregulated bile acids in P4-P30 compared to P30-P60 mice treated with vehicle.

D, E Principal component analysis (PCA) (D) and heatmap (E) of liver metabolomic profile from P4-P30 (green samples) and P30-P60 (red samples) mice transiently treated with vehicle and analyzed at P350. F, female; M, male.

 © 2022 The Authors

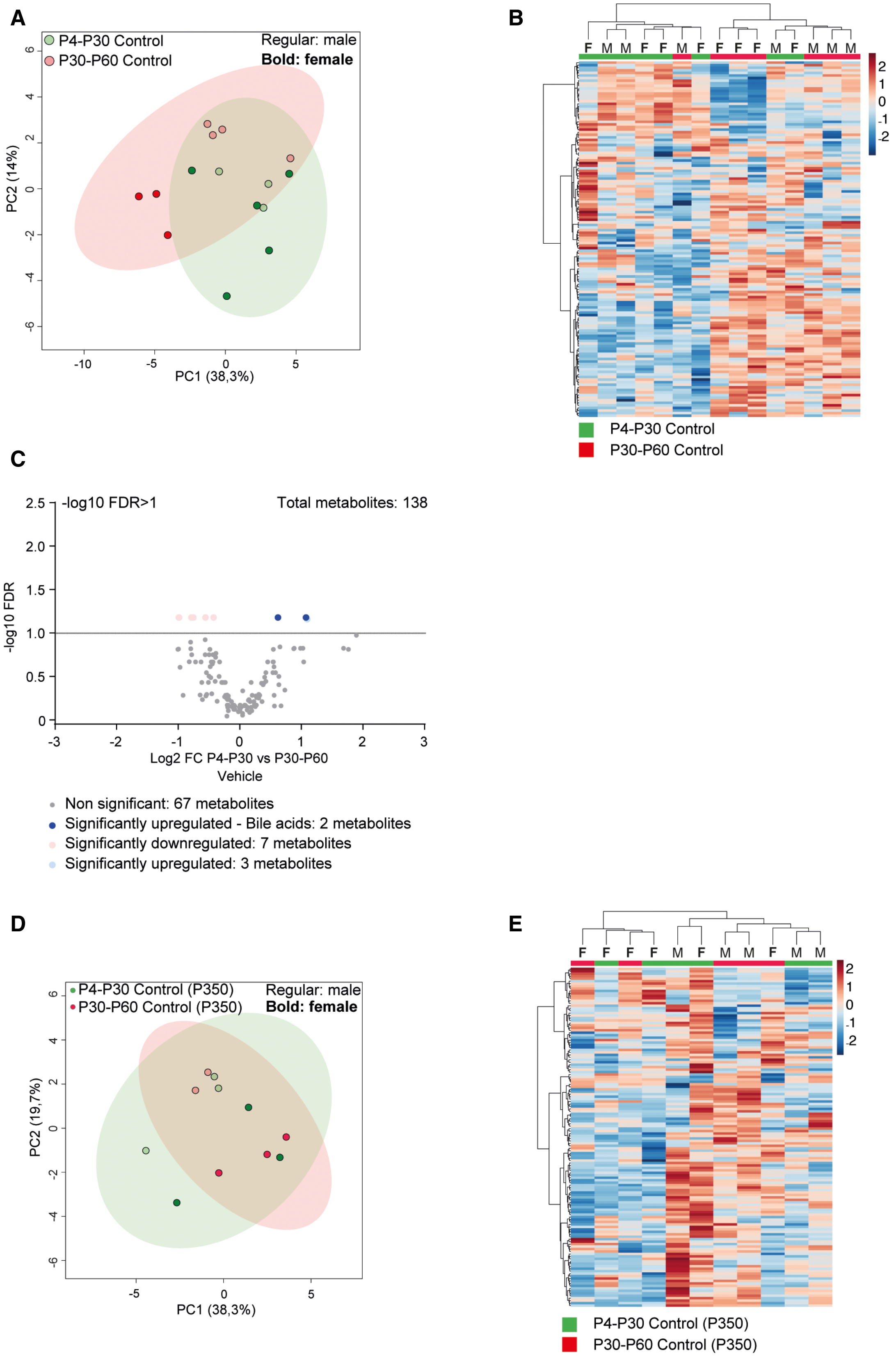
A
P4-P30 Control
P30-P60 Control
Regular: male
Bold: female
PC2 (14%)
PC1 (38,3%)
B
P4-P30 Control
P30-P60 Control
C
-log10 FDR>1
Total metabolites: 138
-log10 FDR
Log2 FC P4-P30 vs P30-P60
Vehicle
Non significant: 67 metabolites
Significantly upregulated - Bile acids: 2 metabolites
Significantly downregulated: 7 metabolites
Significantly upregulated: 3 metabolites
D
P4-P30 Control (P350)
P30-P60 Control (P350)
Regular: male
Bold: female
PC2 (19,7%)
PC1 (38,3%)
E
P4-P30 Control (P350)
P30-P60 Control (P350)

Figure EV3.

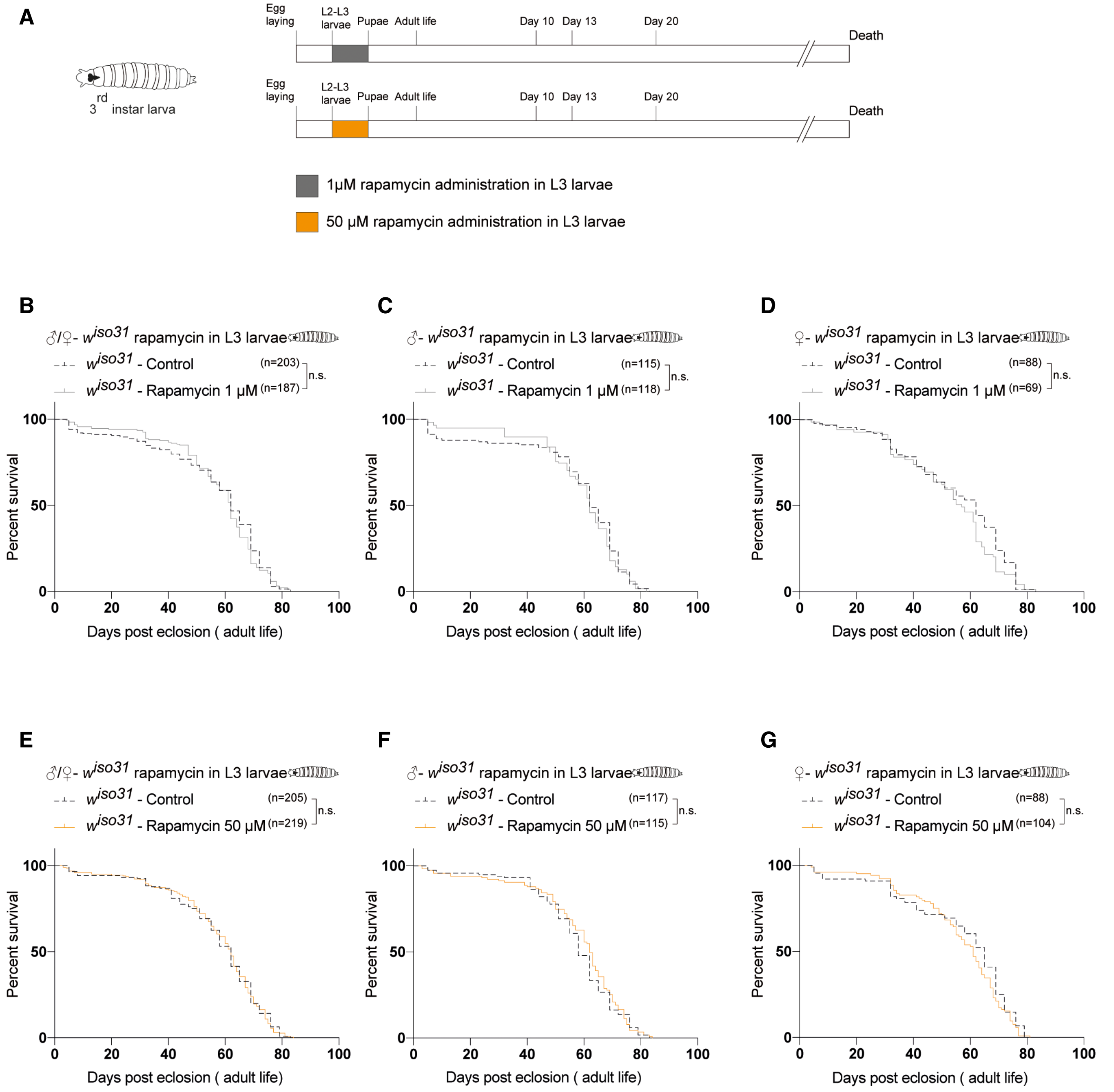

Figure EV4. Early-transient 1 μM and 50 μM rapamycin treatment does not induce a time-dependent effect on lifespan.

A Schematic illustration of the experimental procedure and results. Flies were transiently treated during larval stages from 72 h after egg laying to puparium formation with 1 μM (gray) or 50 μM rapamycin (yellow).

B Survival curves of w^{iso31} flies transiently treated from 72 h after egg laying until puparium formation (males + females) with EtOH (control) or rapamycin 1 μM.

C, D Survival curves of male (C) and female (D) w^{iso31} flies transiently treated from 72 h after egg laying until puparium formation with EtOH (control) or rapamycin 1 μM.

E Survival curves of w^{iso31} flies transiently treated from 72 h after egg laying until puparium formation (males + females) with EtOH (control) or rapamycin 50 μM.

F, G Survival curves of male (F) and female (G) w^{iso31} flies transiently treated from 72 h after egg laying until puparium formation with EtOH (control) or rapamycin 50 μM.

Data information: Log-rank (Mantel–Cox) test; n.s., not significant.

 © 2022 The Authors

Figure EV5. Physical status and biochemical effectiveness of rapamycin treatment in w^{iso31} *Drosophila melanogaster*.

A Representative images showing rapamycin effects on *Drosophila* body size during and after treatment on third-instar (L3) larvae. Images acquired during L3 larvae (120 h after egg laying), pupae, and adult (1 day post-eclosion) stages. Scale bar: 3 mm.

B Representative images showing rapamycin effects on *Drosophila* body size after treatment on 10-day-old flies. Images acquired during adult stage (3 days post-treatment). Scale bar: 3 mm.

C Western blot analysis and quantification of S6 ribosomal protein and phospho-T398-S6 ribosomal protein on whole-fly protein extracts of L3 larvae (left side) and 10-day-old flies treated for 3 days (right side). Two-tailed Student's *t*-test; $^*P < 0.05$.

D, E Gene expression analysis via qRT–PCR of *dST1*(A) and *dST3*(B) in three biological replicates of w^{iso31} L3 wandering larvae ($n = 30$ per biological replicate) and 10-day-old flies ($n = 10$ per biological replicate) treated with 200 μM rapamycin, 12 h after treatment. Data are indicated as mean + SEM. Two-tailed Student's *t*-test; $^*P < 0.05$, $^{***}P < 0.0005$, n.s., not significant.

© 2022 The Authors

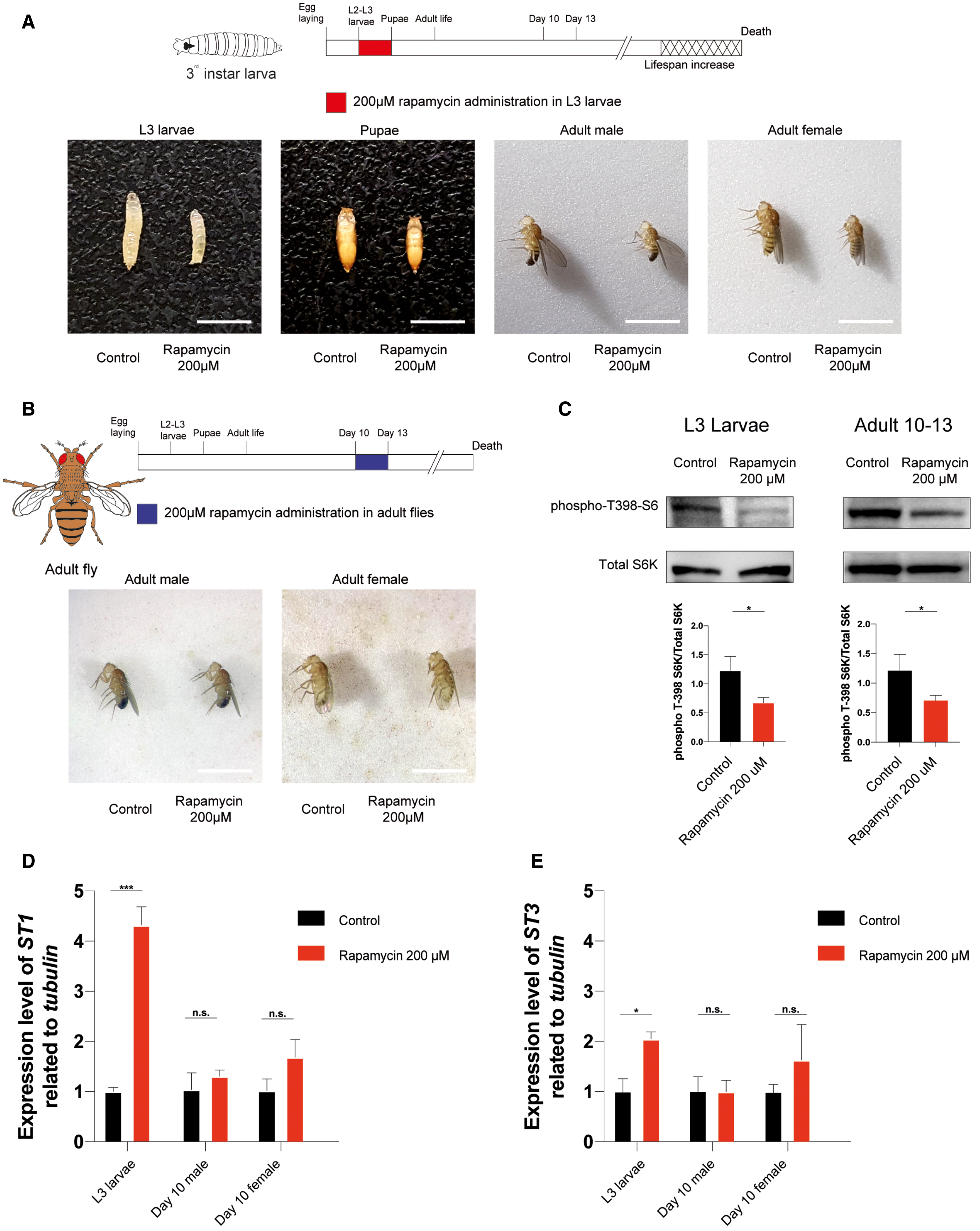

Figure EV5.

© 2022 The Authors
